# Analysis of biofilm assembly by large area automated AFM

**DOI:** 10.1038/s41522-025-00704-y

**Published:** 2025-05-08

**Authors:** Ruben Millan-Solsona, Spenser R. Brown, Lance Zhang, Sita Sirisha Madugula, HuanHuan Zhao, Blythe Dumerer, Amber N. Bible, Nickolay V. Lavrik, Rama K. Vasudevan, Arpan Biswas, Jennifer L. Morrell-Falvey, Scott Retterer, Martí Checa, Liam Collins

**Affiliations:** 1 Center for Nanophase Materials Sciences, Oak Ridge National Laboratory, Oak Ridge, TN 37831 USA; 2 Biosciences Division, Oak Ridge National Laboratory, Oak Ridge, TN 37831 USA; 3 Bredesen Center for Interdisciplinary Research, University of Tennessee, Knoxville, USA; 4 University of Tennessee-Oak Ridge Innovation Institute, Knoxville, TN 37996 USA

**Keywords:** Biological techniques, Antimicrobials, Bacteria, Biofilms, Cellular microbiology, Pathogens

## Abstract

Biofilms are complex microbial communities critical in medical, industrial, and environmental contexts. Understanding their assembly, structure, genetic regulation, interspecies interactions, and environmental responses is key to developing effective control and mitigation strategies. While atomic force microscopy (AFM) offers critically important high-resolution insights on structural and functional properties at the cellular and even sub-cellular level, its limited scan range and labor-intensive nature restricts the ability to link these smaller scale features to the functional macroscale organization of the films. We begin to address this limitation by introducing an automated large area AFM approach capable of capturing high-resolution images over millimeter-scale areas, aided by machine learning for seamless image stitching, cell detection, and classification. Large area AFM is shown to provide a very detailed view of spatial heterogeneity and cellular morphology during the early stages of biofilm formation which were previously obscured. Using this approach, we examined the organization of *Pantoea* sp. YR343 on PFOTS-treated glass surfaces. Our findings reveal a preferred cellular orientation among surface-attached cells, forming a distinctive honeycomb pattern. Detailed mapping of flagella interactions suggests that flagellar coordination plays a role in biofilm assembly beyond initial attachment. Additionally, we use large-area AFM to characterize surface modifications on silicon substrates, observing a significant reduction in bacterial density. This highlights the potential of this method for studying surface modifications to better understand and control bacterial adhesion and biofilm formation.

## Introduction

Biofilms are multicellular communities of microbial cells held together by self-produced extracellular polymeric substances (EPS) which also help them to adhere to biotic or abiotic surfaces^1^. Biofilms are ubiquitous in natural, industrial, and clinical environments, playing critical roles in various ecosystems while also posing significant challenges in healthcare due to their resilience against antibiotics and disinfectants^2,3^. Although biofilms have been recognized since van Leeuwenhoek’s 17th-century observations, our understanding of the mechanisms driving biofilm assembly, persistence and their roles in biofilm resilience to environmental stresses is incomplete and hinders the development of effective strategies for manipulating biofilm growth^4^. This lack of understanding arises partly from the inherent heterogeneous and dynamic nature of biofilms, characterized by spatial and temporal variations in structure, composition, density, metabolic activity, and microenvironmental conditions^5^. These variations are in turn influenced by microbial species, environmental conditions, and surface properties, as well as microbial interactions, nutrient gradients, and the production of EPS, all of which contribute to biofilm stability, resilience, and resistance to external stressors^6,7^. On top of this, traditional analytical methods often fail to capture the full scope of this complexity, failing to link local subcellular and cellular scale changes to the evolution of larger functional architectures. This underscores the necessity for advanced imaging techniques that enable comprehensive biofilm characterization across these scales^8^.

A range of analytical methods has been utilized to study the structure and organization of biofilms, each presenting unique advantages and limitations that have been reviewed elsewhere^9,10^. For example, light microscopy permits basic morphological observations but suffers from low resolution. Confocal laser scanning microscopy provides higher-resolution, three-dimensional images of biofilms but requires fluorescent staining of cells or biomolecules, which may alter the inherent properties of biofilms. Scanning electron microscopy offers detailed surface imaging but involves sample dehydration and the application of metallic coatings, potentially distorting microbial structures^11^. Spectroscopic techniques such as Fourier transform infrared spectroscopy can identify chemical constituents but often lack spatial resolution, while Raman spectroscopy delivers detailed chemical information but is limited by fluorescence interference and necessitates the use of high laser powers which can cause photodamage.

High-resolution imaging techniques are imperative for studying cell structures, cell-to-cell interactions, and finer features like cell walls and appendages, such as outer membrane extensions^12^ flagella and pili^13^. The key challenge is to simultaneously capture both large-scale architecture and the finer cellular features^14^ of interest, enabling the investigation of structural and functional responses at the sub-cellular level. This capability would allow us to study how cells attach to surfaces, develop into complex communities with physical, chemical, and functional heterogeneity, and respond to external stresses. Atomic force microscopy (AFM)^15^ enables the imaging of cell structures, interactions, and mechanical properties at the nanoscale without extensive sample preparation, and it can even be used under physiological conditions^16–18^. By scanning a sharp probe over the surface and measuring the forces between the probe and the sample, AFM provides nanometer-scale topographical images as well as quantitative maps of nanomechanical properties^19^. This allows AFM to reveal structural features that surpass the resolution of optical or electron beam-based microscopy^20,21^. For instance, AFM can offer detailed insights into bacterial cells, highlighting membrane protrusions, surface proteins, and cell wall ridges^22^. Fine structures, such as the EPS that form the biofilm matrix, can also be visualized with high clarity. These structures include polysaccharides, proteins, and nucleic acids that bind the biofilm together^23^.

High-resolution imaging not only reveals how these components interact to provide structural stability and protection to the bacterial community but also contributes to the development of targeted interventions. Additionally, when operated in liquids, AFM preserves the native state of cells or microbes and can measure mechanical properties^24^ like stiffness, adhesion, and viscoelasticity, or electrical properties like dielectric constant^25^, which has recently been shown to enable the extraction of internal characteristics from biological samples^26–29^, offering deeper insights into structure–function relationships. AFM has not only proven to be a powerful tool for nanoscale mechanical and topographical characterization, but it also enables the integration of various non-invasive chemical imaging^30,31^ and composition mapping^32^, internal hydration properties of single bacterial endospores^33^, and properties of outer membrane extensions of bacteria^12^.

While AFM provides detailed structural and mechanical insights in biology and cell research, its impact on biofilm research has been surprisingly limited. Conventional image analysis methods allow for the measurement of individual cell dimensions, but automated techniques are crucial for efficiently characterizing bacterial biofilms, which can contain up to 10^12^ cells per gram. AFM’s small imaging area (<100 µm), restricted by piezoelectric actuator constraints, limits its capacity to study large, millimeter-scale biofilm structures. This scale mismatch makes it difficult to capture the full spatial complexity of biofilms and raises questions about the representativeness of the collected data. Furthermore, the slow scanning process and labor-intensive operation require specialized operators, hindering the capture of dynamic structural changes over extended time and length scales^34^. Developing automated large-area AFM methods and integrating AFM with other multimodal techniques will significantly advance biofilm research by enabling comprehensive analysis of these complex microbial communities^35^.

Machine learning (ML) and artificial intelligence (AI) are transforming AFM by enhancing data acquisition, control, and analysis. ML applications in AFM fall into four key areas: sample region selection, scanning process optimization, data analysis, and virtual AFM simulation^36^. AI-driven models optimize scanning site selection^37^, reducing human intervention and accelerating acquisitio^38,39^ ML can be further used to improve scanning by refining tip–sample interactions^40^, correcting distortions^41^, reducing the time by sparse scanning approaches^42,43^, and automating probe conditioning^44^ for more precise imaging. AI frameworks have also enabled autonomous operation^45^ of scanning AFM and direct control of through large language models^46^, not by replacing human operators but by automating routine tasks, optimizing decision-making processes, and enhancing scientific discovery through human–ML collaboration and enabling allowing continuous even multiday experiments without human supervision^14,47^. In data analysis of AFM, ML, and AL tools are enabling automated segmentation, classification, and defect detection in AFM images^48^, aiding in cancer cell^49,50^ identification^51^ and molecular structure prediction^52^. These advancements significantly enhance AFM’s efficiency, accuracy, and automation, particularly in biological research and nanomaterials characterization. For a deeper understanding, readers can refer to recent reviews on ML and AI applications in AFM^36,53^.

In this work, we address the challenges of biofilm imaging by AFM through the development of an automated large-area AFM which is capable of analyzing microbial communities over extended surface areas with minimal user intervention. By automating the scanning process, we overcome many limitations of traditional AFMs, including small imaging areas, and enable imaging of the inherent millimeter-sized communities. We evaluate image stitching algorithms for performance with minimal matching features between images. Limited overlap between scans maximizes acquisition speed, producing seamless, high-resolution images that capture the spatial complexity of surface attachment. To manage the high-volume, information-rich data, we implement machine learning-based image segmentation and analysis methods. These tools assist in automating the extraction of important parameters, such as cell count, confluency, cell shape, and orientation, and facilitate efficient and quantitative analysis of microbial community characteristics over extensive areas. We demonstrate the applicability and effectiveness of our methods by imaging the initial surface attachment of *Pantoea* sp. YR343. Additionally, we apply large-area AFM to gradient-structured surfaces, allowing us to study how varying surface properties influence attachment dynamics and community structure in a combinatorial approach. By integrating these advancements, our work enhances the capabilities of AFM for biofilm research, enabling comprehensive structural and mechanical characterization of biofilms at scales relevant to their natural environments.

## Results

### High-resolution AFM imaging of *Pantoea* sp. YR343

In this study, we focus on *Pantoea* sp. YR343, a gram-negative bacterium isolated from the poplar rhizosphere, is known for its plant-growth-promoting properties. *Pantoea* sp. YR343 is a rod-shaped, motile bacterium with pili and peritrichous flagella, which facilitate its interactions within its environment. This strain forms biofilms on plant roots and on abiotic surfaces and mutants have been identified that are defective in the process of biofilm formation and root colonization^54,55^.

**Fig. 1:**
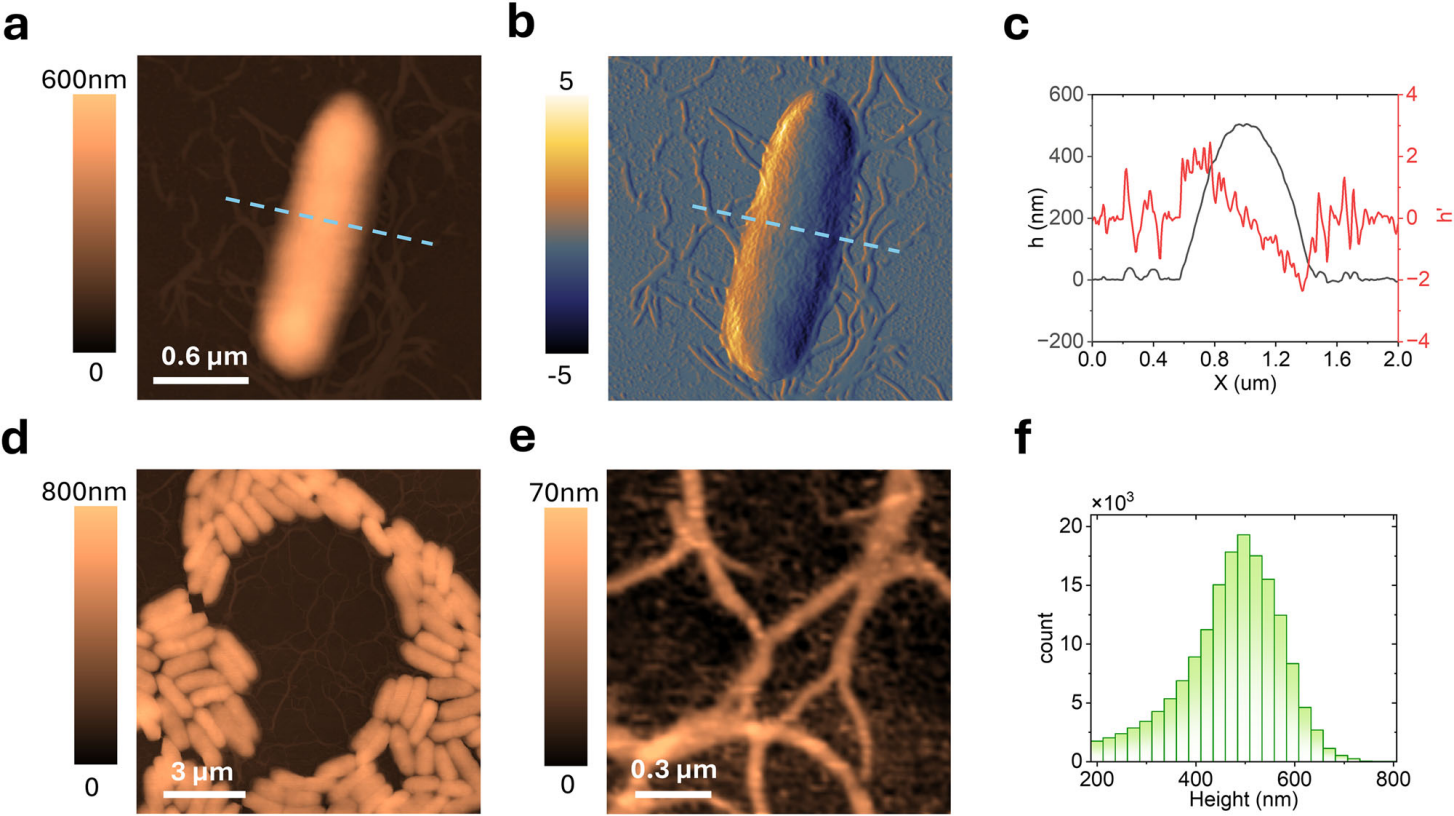
High-resolution AFM imaging of *Pantoea* sp. YR343. **a** Topography and **b** derivative image of a single cell, showing filamentous structures and appendages deposited on the surface. **c** Cross-section of the area marked by the dashed line in (**a**) and (**b**). **d** Topography image of a cluster of cells and **e** a zoomed-in view of flagellar structures extending across the gap. **f** Histogram of height measurements taken from (**d**).

To observe surface attachment dynamics and biofilm growth, a petri dish containing PFOTS-treated glass coverslips was inoculated with *Pantoea* cells growing in a liquid growth medium. At selected time points, a coverslip was removed from the Petri dish, gently rinsed to remove unattached cells, and dried before imaging as described in the “Methods” section. As shown in Fig. 1a–c, surface-attached *Pantoea* cells observed after a brief incubation (~30 min) were typically around 2 µm in length and 1 µm in diameter, corresponding to a surface area of ~2 μm², aligning with previous findings^56^. AFM imaging provided structural details not achievable with optical microscopy or other methods, enabling visualization of flagellar structures around the cells, measuring ~20–50 nm in height and extending tens of micrometers across the surface. Some appendages appeared to originate from individual cells, while others seemed to adhere to the surface, possibly from detached cells. The identification of these structures as flagella was confirmed using a flagella-deficient control strain, which showed no similar appendages under AFM (see Supplementary Fig. 1 in the Supplementary Information).

In Fig. 1d, cells allowed to propagate on the surface for a period of 6–8 h formed clusters with characteristic honeycomb-like gaps^56^. AFM’s high-resolution capability allowed clear visualization of individual cells and flagella (see Fig. 1e). In *Pantoea* sp. YR343, AFM revealed flagellar structures bridging gaps during early cell attachment and development. These detailed visualizations are critical, as appendages like flagella are essential for biofilm development, surface attachment, and motility. Without high-resolution imaging, such structural intricacies would remain hidden, limiting our understanding of biofilm assembly. Flagella may also increase the surface area for attachment, guiding nascent cells and providing additional points of contact across biofilm gaps. Previous studies using mutant strains lacking flagella showed significantly reduced surface coverage, highlighting the role of flagella in biofilm assembly and structure^56^.

The primary challenge for more meaningful biofilm research by AFM is simultaneously capturing these fine cellular or sub-cellular structural and functional details across the much larger-scale community architecture. Achieving this would allow us to explore how subtle changes in sub-cellular and cellular structure influence the overall organization, physiology, and function of the developing biofilm. A secondary challenge is simplifying the analysis process. While images like Fig. 1d enable simultaneous height measurements of multiple bacteria through pixel height distribution analysis (see histogram in Fig. 1f), measuring parameters such as lateral dimensions, eccentricity, and orientation remains labor-intensive. This becomes a major bottleneck when attempting to analyze cell properties across the entirety of a biofilm. In the following sections, we present an automated workflow for large-area AFM acquisition and analysis, enhancing statistical power and enabling the observation of microbial surface attachment and biofilm growth and structure over time. This multiscale approach bridges microscopic details and macroscopic properties in an automated fashion, ultimately enhancing our ability to study and manage biofilms effectively.

### Automated workflow for large-area AFM acquisition

**Fig. 2:**
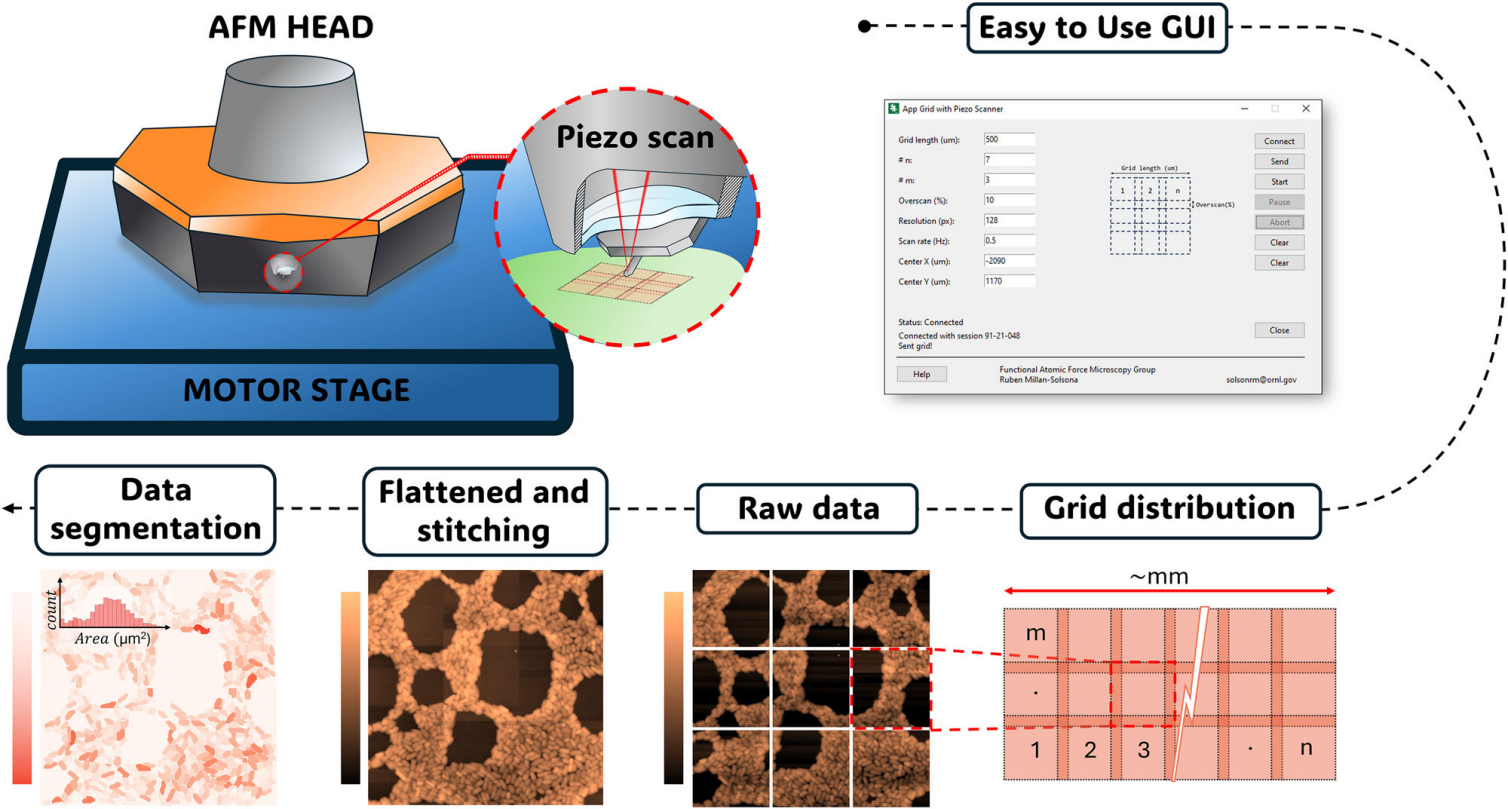
Schematic of automation protocol and example of large-area imaging. The large-area AFM setup presented here includes an automated AFM imaging platform with a user-friendly graphical interface for configuring optimal grid design and spacing. The system also features an automated processing and analysis workflow, which involves image flattening, stitching, and segmentation to generate property maps and perform statistical analyses.

To achieve this, we have developed and implemented a large-area AFM using a commercial DriveAFM microscope (Nanosurf) equipped with an automated sample stage, as illustrated in Fig. 2. Stage positioning and imaging parameters—including the number of pixels, scan rate, scan region size, grid image overlap, and the total number of images—were controlled through a graphical user interface (GUI) operated via custom Python functions. Once the user-defined parameters were set, the GUI interacted with the microscope to capture multiple grid images automatically over a predefined area of the sample. After data acquisition, the individual images underwent flattening procedures and were stitched together into a seamless mosaic image, as discussed in the following section. This composite image was then ready for image segmentation and analysis. This user-friendly, automated approach eliminates many of the manual tasks that previously made large-area AFM of biofilms impractical, significantly enhancing efficiency and usability. The code used for automation and image processing is available in the “Code availability” section, enabling other researchers to replicate and build upon our work.

Quantifying biofilm structural properties and dynamics involves measuring specific metrics, such as cell count, confluency, height/layer number (i.e. biomass), along with the production of EPS and structural characteristics like surface roughness. These comprehensive measurements and analyses provide key information for understanding biofilm dynamics and aid in the development of effective biofilm management strategies. However, to accurately extract statistically relevant quantitative descriptors from the large area AFM images, it is first necessary to explore image stitching algorithms, with improved performance at minimal matching features or overlapping images. These algorithms ensure that the individual high-resolution images are seamlessly merged to form a coherent and continuous representation of the entire biofilm. Accurate stitching is necessary to preserve the spatial relationships and continuity of structural features, allowing for a true representation of the biofilm’s architecture and organization. In addition, precise image stitching minimizes distortions and artifacts that could lead to misinterpretations or errors in measurements of the biofilm’s physical properties (e.g. cell count, confluency, etc.).

**Fig. 3:**
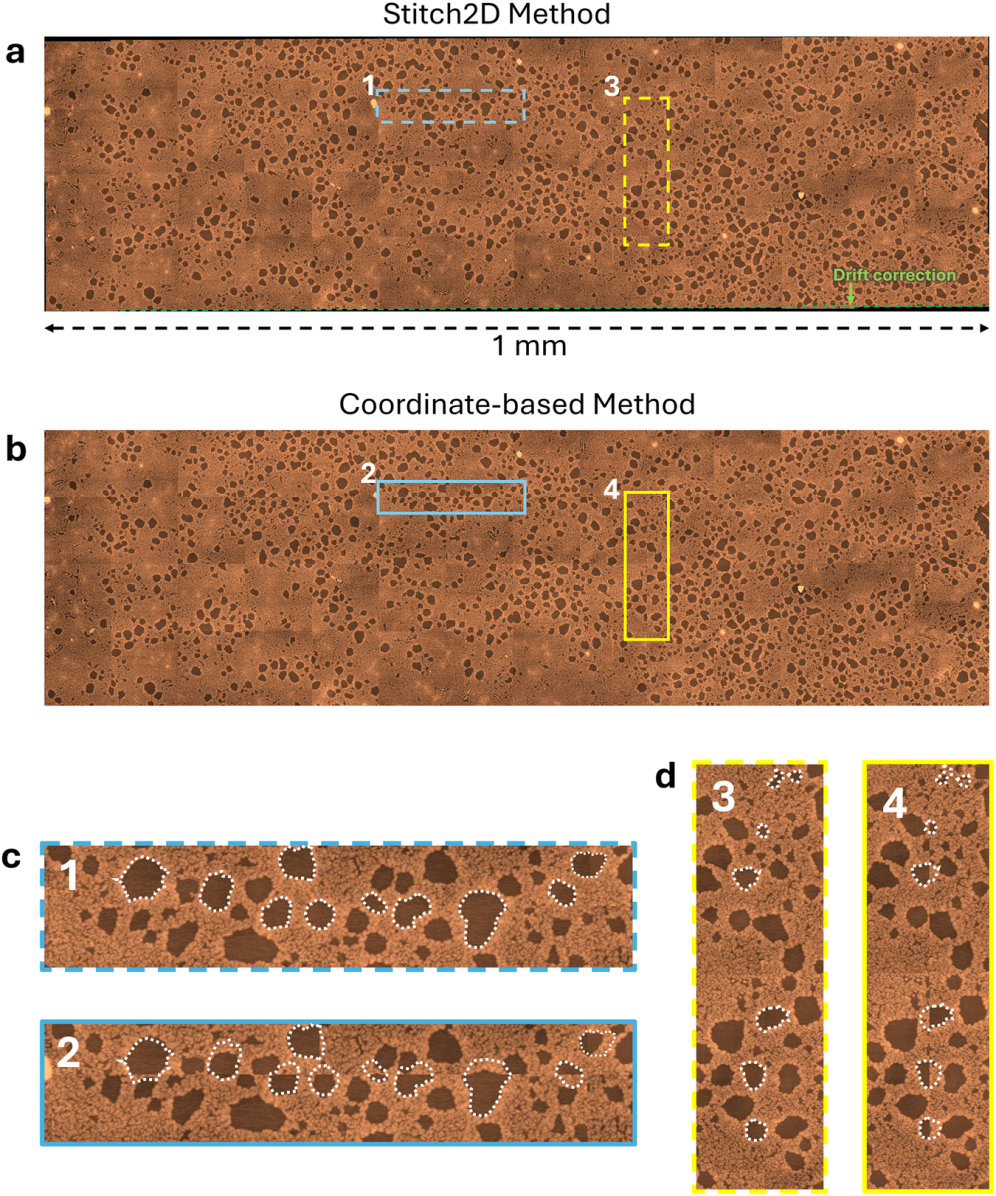
Large area stitched image. **a** Large-area image of surface-attached *Pantoea* sp. YR343 cells are stitched using the Stitch2D algorithm, which incorporates the SIFT feature detection method. **b** Image stitched using the coordinates provided by the AFM scanner, showing drift and alignment issues. **c** Close-up horizontal sections and **d** close-up vertical sections from (**a**, **b**). Show how the Stitch2D algorithm effectively reconstructs the image and removes obvious stitching artifacts when the coordinate system along was used (white dashed lines) due to significant errors due to misalignment and scanner drift, leading to repeated or missing features. This analysis highlights the importance of accurate image stitching to produce seamless, high-resolution representations of community structure, necessary for reliable downstream analysis such as segmentation and morphological assessments.

Figure 3 illustrates the performance of two image stitching algorithms applied to AFM-generated images of surface-attached bacterial cells. When considering stitching methods, we recognize that various stitching approaches exist, each with strengths and limitations^57–59^. Direct methods, like Lucas–Kanade optical flow and the sum of absolute differences (SAD), align images using pixel intensities but can struggle with large displacements or illumination changes. Transform-based techniques, such as Fourier transform-based phase correlation, are faster and noise-resistant but have issues with rotation and scaling. Multi-scale approaches, such as pyramid-based stitching, improve robustness by refining alignment progressively, making them suitable for larger images with significant variations. Feature-based methods like scale-invariant feature transform (SIFT)^60^ and speeded-up robust features (SURF)^60^ detect and match key points across overlapping images, with SIFT excelling in robustness and SURF optimized for speed. For real-time applications, Oriented FAST and rotated BRIEF (ORB) offer computational efficiency but handle scale variations less effectively than SIFT^61^. According to a recent comparative analysis^62^ of pairwise image stitching techniques for microscopy images, SURF emerges as the most effective feature detector. While SURF provides better performance than SIFT, it poses implementation challenges due to its licensing restrictions. The choice of method depends on factors such as image characteristics, computational resources, and required accuracy. Combining techniques and integrating machine learning can further enhance stitching performance, ensuring accurate large-scale, high-resolution image analysis.

In Fig. 3a, we used the Stitch2D algorithm, which uses SIFT as a feature detector, while Fig. 3b displays the results when using coordinate-based alignment from the AFM scanner. Both methods aim to merge individual high-resolution scans into a seamless large-area image. In Fig. 3c and d, the challenges of drift correction and stitching errors are highlighted. Scanner drift is traditionally the major and unavoidable source of artifacts exhibited in AFM images^63^. Stitch2D effectively reconstructs the large-area AFM image, successfully reducing errors caused by scanner drift and overlap artifacts (as shown in the white dashed lines). The significance of precise stitching for large-area AFM imaging is underscored, particularly for complex communities where accurate representation is crucial for downstream analyses such as cell segmentation and morphological assessment.

**Fig. 4:**
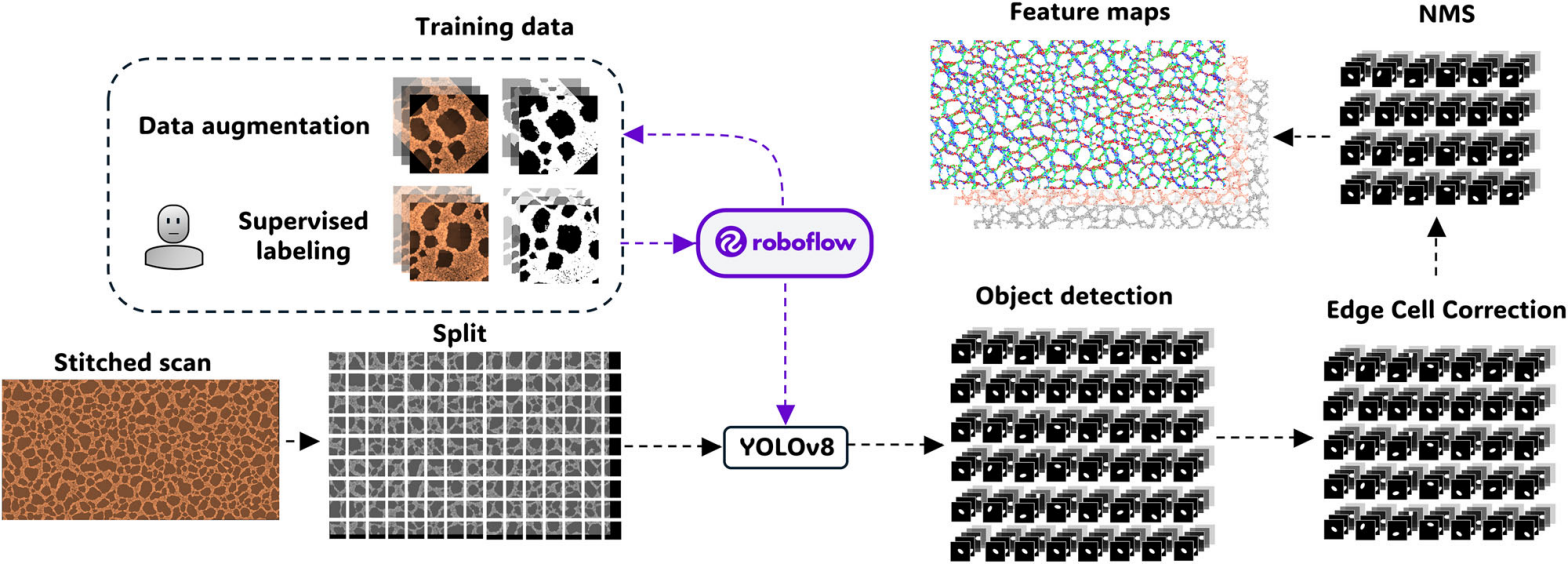
Workflow schematic of the image analysis. Top left: How a small manually labeled dataset is expanded with Roboflow to train a YOLOv8 model. Bottom left: The large area map is split into overlapping 15 × 15 µm² regions, which are analyzed by the model, producing a mask for each detected bacterium. To remove duplicates from overlap and edge effects, we apply a filter and global non-maximum suppression. Final masks are used to analyze bacterial properties for individual cells which are tabulated and used to generate feature maps.

Figure 4 illustrates the image segmentation workflow developed for analyzing AFM images of surface-attached microbial communities using the YOLOv8 model. Image segmentation is a computer vision technique that partitions an image into distinct regions or objects, accurately delineating their boundaries^64^. The YOLOv8 model, the latest in the “You Only Look Once” series, represents the state-of-the-art in object detection and image segmentation, excelling in tasks requiring precise localization and boundary identification. YOLO models are renowned for their speed and accuracy, making them widely applicable across fields, including biomedical imaging^65–68^. In this study, the YOLOv8 model is instrumental in accurately identifying bacterial cells and extracting their physical features from AFM images. This workflow begins with a preliminary set of standard 15 × 15 µm² AFM images, which are used for supervised labeling on the Roboflow platform^69^. This platform facilitates the tasks of labeling, segmentation, and data augmentation through techniques such as rotation and resizing (see Supplementary Fig. 2). Additionally, Roboflow enables the division of the dataset into training, validation, and test sets in a 70–20–10 ratio and optimizes the efficiency and speed for training these types of models.

Once a YOLOv8 model with sufficient accuracy is obtained, it is used to segment a large-area stitched image (see Supplementary Fig. 3 for details on hyperparameter optimization). To do this, the image is divided into smaller images of the same size as those used in training, with a 10% overlap between them. This overlap reduces the risk of segmentation errors at the edges of the sub-images. Segmenting these sub-images produces a mask for each detected bacterium in every image, generating a large volume of data.

Next, an edge correction filter is applied to eliminate masks of objects that are touching or very close to the image edge. Then, given that the sub-images overlap, the non-maximum suppression (NMS) method is used to remove duplicate objects, ensuring that only the object with the highest score is kept, thus avoiding the duplication of bacteria in overlapping areas.

The final step involves calculating properties for each detected bacterium, including area, eccentricity, major axis, minor axis, and orientation, and then creating a map for each of these properties. This structured workflow ensures efficient data preparation and accurate bacterial segmentation through machine learning techniques. An example of a data set can be seen in Supplementary Fig. 4. Overall, this workflow enables automated and comprehensive analysis of the community structure, providing high-resolution metrics at the cellular level and facilitating a deeper understanding of biofilm assembly and growth patterns^4^.

### Large-area AFM imaging of *Pantoea* sp. YR343 biofilms on PFOTS-treated surfaces

**Fig. 5:**
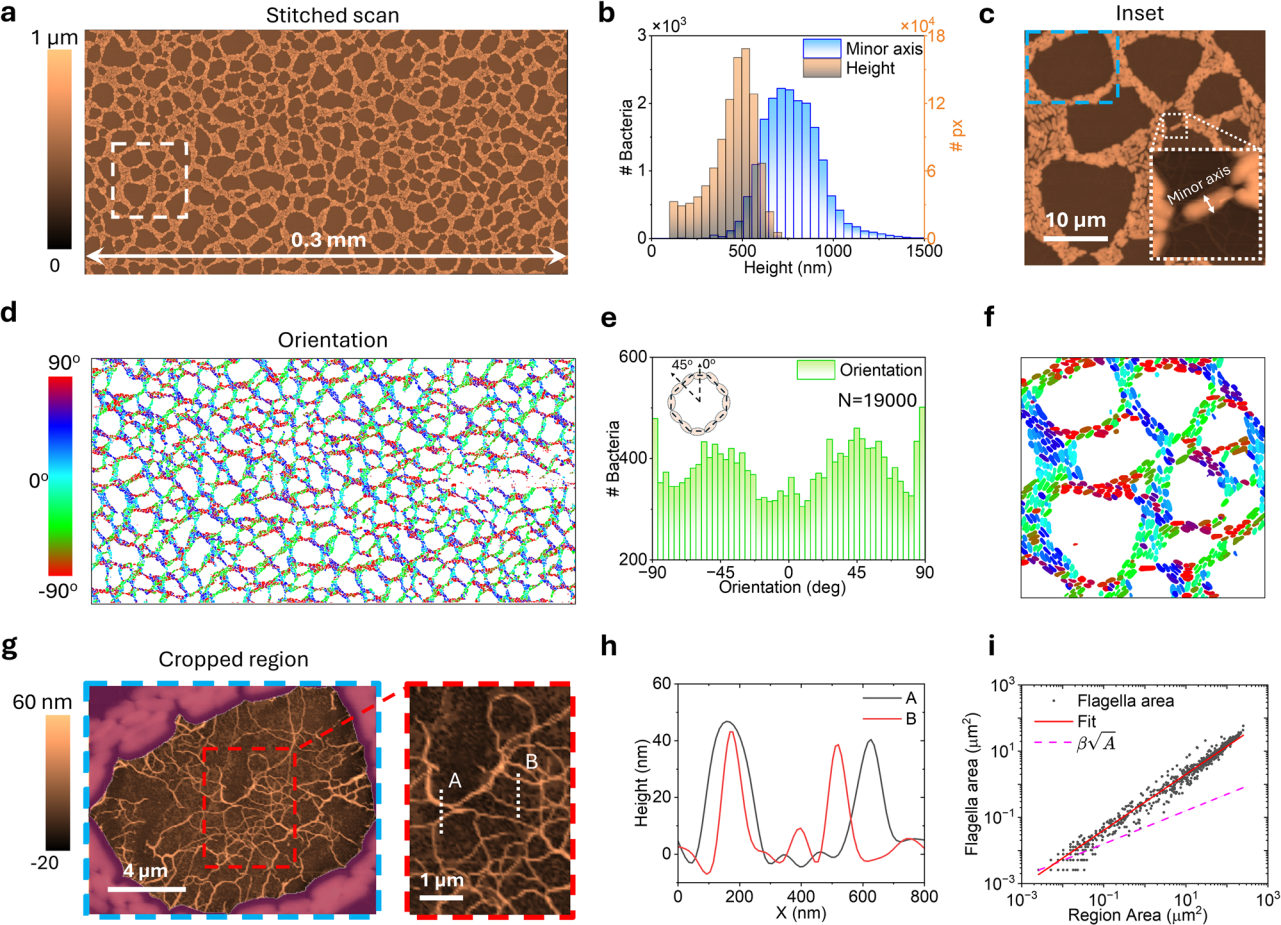
Automated analysis of microbial community structure using large-area AFM data. Top row: Height map of surface-attached cells with corresponding **b** height and minor axis distribution histogram and **c** zoomed-in region marked in (**a**). **d** Large orientation map indicating the directional alignment of cells, **e** orientation distribution histograms and **f** zoom-in regions that highlight the orientation of the cells (**a**), **g** example of a cropped region highlighting the flagella corresponding to the region marked by the blue box in (**c**). **h** shows profile height marked in the red box in (**g**). **i** The dependence of the area covered by the flagella vs. the area of the region of the entire image in (**a**) is shown. In red, the fit of the data to the model and dashed magenta shows ${A}_{{\rm{f}}}\propto \sqrt{A}$ dependency.

Figure 5 demonstrates the application of our developed methodology on *Pantoea* sp. YR343 was grown on PFOTS-treated glass surfaces for a growth period ranging between 6 and 8 h, covering an area of 0.3 × 0.15 mm². When working at this larger millimeter scale, acquisition times can range from several hours to multiple days, depending on the scanned area and resolution. For instance, the scanning time for Fig. 5 was ~16 h providing a 6056 × 3064 px^2^ stitched image. However, this extended acquisition allows us to visualize the pattern of initial surface attachment by *Pantoea sp*. YR343 cells. Similar to previous reports^56^, the large area AFM reveals a honeycomb-like structure with linear branches of cells intersecting to form a net-like appearance, creating gaps that are gradually segmented and eventually filled as the community grows (see Fig. 5a). In future work, we are exploring the implementation of sparse imaging techniques and adaptive scan paths^43^ to significantly reduce acquisition time, which is essential for studying biofilm assembly dynamics in real-time.

In Fig. 5b, thanks to automation, it is possible to compare the height distribution of each bacterium with the distribution of its minor axis (see inset in Fig. 5c). The difference between these two measurements is likely due to the flattening^70^ of the bacteria upon adhesion to the surface, a commonly observed phenomenon caused by strong membrane–surface interactions. Additionally, the effects of tip convolution contribute to a broadening of some tens of nanometers, with this effect being more pronounced in smaller structures such as flagella. Together with the measurement of cellular eccentricity or area (see Supplementary Fig. 5), this allows us to identify how stretched or irregularly shaped the cells are, which in turn can correlate with certain functional adaptations or responses to environmental conditions. In this case, cellular eccentricity showed a normal distribution with a mean of 0.8 ± 0.1, as did the average area per bacterium of 0.96 ± 0.4 µm². Some cells as large as 2 µm² were also observed that were likely in the process of cell division (see Supplementary Fig. 5). These values were obtained from analyzing 19,000 cells in this region. This extensive dataset permitted spatial analysis of cell clustering, as shown in Supplementary Fig. 6, which revealed weak but statistically significant global spatial autocorrelation (Moran’s *I*), indicating some clustering of similar cell sizes. Additionally, *K*-means clustering suggests that cells exhibit some grouping by area. However, the radial autocorrelation function indicates that spatial dependency quickly diminishes with distance, suggesting localized clustering at small scales but a largely random distribution beyond a certain radius.

The orientation maps (Fig. 5d–f) reveal a preferred alignment of cells at approximately -45° and 45°, suggesting a highly organized structural pattern during surface attachment. This alignment likely plays a role in the formation of the microbial community’s honeycomb-like structure by promoting regular, repeating patterns that enhance stability and cohesion among cells. Such preferred orientations may allow cells to pack efficiently, minimizing gaps while maximizing surface area for attachment and nutrient exchange. This organized arrangement could also facilitate cooperative interactions between cells, contributing to the resilience and robustness of the biofilm structure. The alternation between −45° and 45° orientations may further support this honeycomb architecture, as it creates interlocking, tightly packed cell clusters that enhance the biofilm’s structural integrity.

These cell segmentation maps provide a wealth of information on cell morphology and positioning, offering insights into the structural organization during biofilm assembly and growth. However, additional details on flagella are also embedded in these maps but remain obscured by the current color scale. Here, we unlock this information to reveal further insights into the spatial distribution and orientation of flagella, which play a crucial role in surface attachment and cell-to-cell interactions. In a previous study by Halsted et al., it was observed that flagella plays a crucial role in biofilm formation and structure. To take a closer look at this process, we performed masked flattening of the gap regions individually. This improves the flattening and facilitates the measurement of the characteristics of the flagella (See Supplementary Fig. 7). As shown in Fig. 5g and h, the flagella of *Pantoea* sp. YR343 extends across gaps in the honeycomb-like structure, likely facilitating cell-to-cell interactions and structural integrity. We observe individual fibrils of ~10–20 nm, which is consistent with reported flagellar dimensions in the literature^71^, in contrast to the expected diameter of pili, which is typically around 5 nm. These fibrils can combine to form larger structures exceeding 40 nm, which likely strengthens surface attachment and connectivity within the community, and possibly plays a role in the overall stability, resilience, integrity, and development of the biofilm. Thanks to the large number of regions available, we can study the relationship between the area covered by flagella and the total area of the selected regions (Fig. 5i). When plotting the data for all these regions on a log-log scale, we observe a linear trend. This suggests a power-law relationship between the area covered by flagella and the total area of the region, which can be expressed as ${A}_{{\rm{f}}}=\beta {A}^{b}$, where the slope of the line gives us the value of the exponent *b*.

In this simplified model (see Supplementary Section 8), if we only considered flagella belonging to bacteria located on the perimeter of each region, we would expect the exponent to be approximately 0.5. This is because, in this case, the amount of flagella would be proportional to the perimeter length (which grows with the square root of the area, $\propto {A}^{0.5}$). In our study, we observe an exponent close to 0.9. This value, notably higher than 0.5, suggests that flagella contributions arise not only from bacteria positioned along the perimeter but also from cells located deeper within the honeycomb structure, as well as from flagella left behind by detached cells. This finding points to the fact that flagella, even in their detached state, may also play a critical role in conditioning the surface, facilitating attachment for additional colonizing cells, and promoting further biofilm expansion. This type of analysis is only possible due to the large number of regions observed by large-area automated AFM. Repeating this study under different conditions, such as various bacterial growth stages, nutrient gradients, or environmental variations, could provide valuable insights into how flagella distribution contributes to the formation and structure of biofilms. This approach would allow us to better understand the organizational patterns and adaptability of bacteria in response to their environment.

### Optimization of structured surfaces for antifouling and biofilm control

Finally, we aim to demonstrate the utility of this approach for studying and optimizing surface modifications and structured surfaces for antifouling and biofilm disruption—an area of active research focused on preventing microbial adhesion and biofilm formation on various surfaces. This research has significant applications in medical, industrial, and marine environments, where biofilms can lead to persistent infections, equipment fouling, and costly maintenance. By introducing specific surface patterns or textures, structured surfaces can physically inhibit microbial attachment and biofilm formation, potentially reducing the need for chemical antimicrobials.

To investigate the effects of structured surfaces on bacterial adhesion, we employ a combinatorial gradient of silicon ridges with variable spacings, from 3 to 20 µm, a height of 1.3 µm, and a 3 µm gap between plateaus. This gradient allows us to assess how varying ridge spacings influence bacterial attachment and community morphology across a continuous spectrum of structural parameters, providing valuable insights for the development of optimized antifouling designs.

**Fig. 6:**
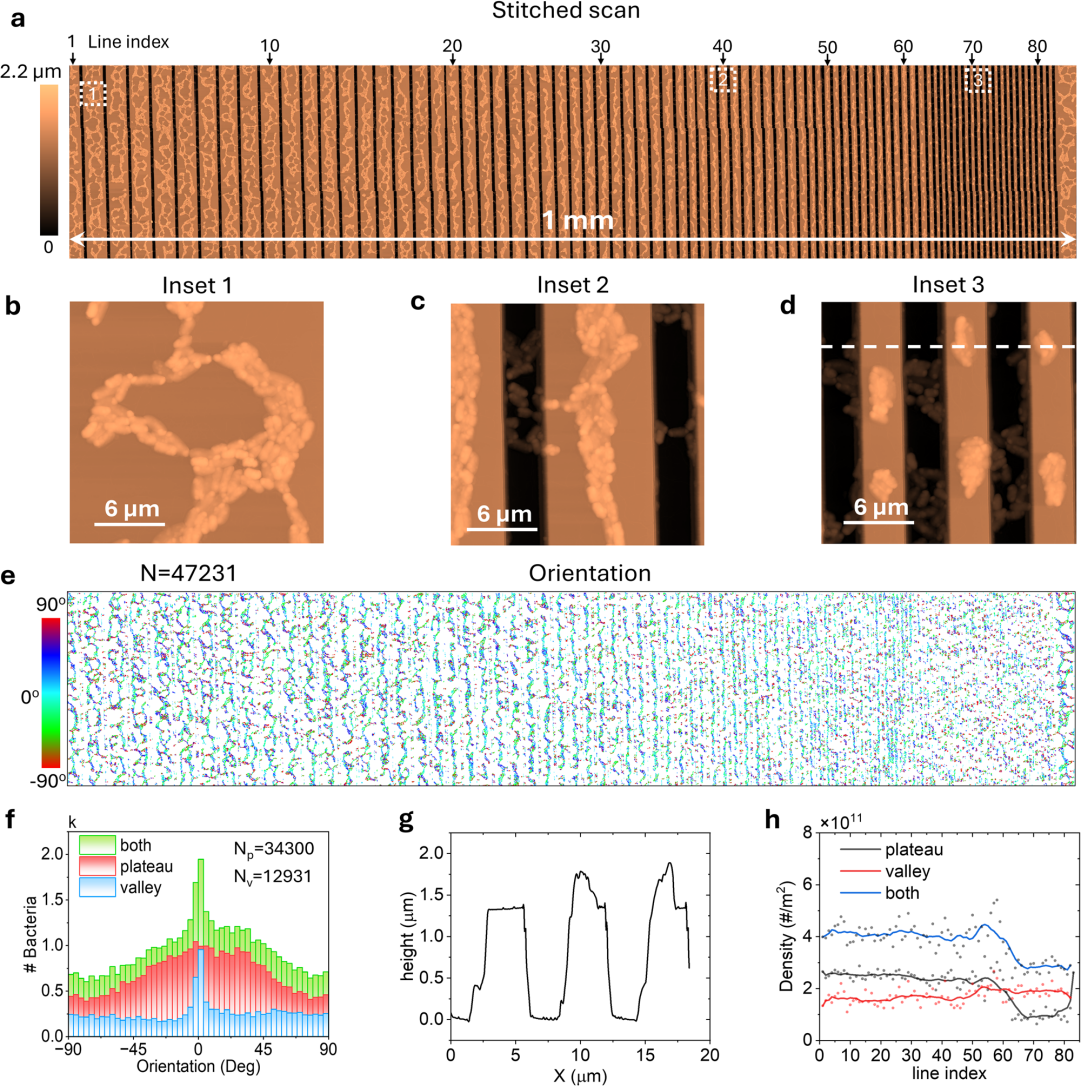
Automated analysis of surface-attachment patterns on structured interfaces using large-area AFM data. **a** Height map of the surface with attached cells. **b**–**d** zoom-in regions highlighting cell area in (**a**). **e** Large orientation map indicating the directional alignment of cells. **f** Histogram of the orientation distribution for the two populations and the sum of both. **g** Height profile marked with a dotted white line in (**e**). **h** Density vs. line index for the two populations and the sum of both are shown.

Figure 6 demonstrates the application of our developed methodology on *Pantoea* sp. YR343 cells were grown for a period of 6–8 h on silicon patterned surfaces treated with PFOTS, which was coated for 60 min, covering an area of 1 × 0.25 mm², with an acquisition time of 135 h and a final resolution of 23,905 × 4583 px^2^. The analysis has allowed us to measure 47,231 bacteria and separately analyze both the bacteria on the plateaus and in the valley of the structured silicon surfaces.

In Fig. 6a, regions outside the grid pattern reveal surface-attached cells forming the characteristic honeycomb structure, as previously discussed. On surfaces with larger ridge spacings, this honeycomb architecture remains largely unaffected; however, an edge effect is evident, confining the honeycomb formations to the center of the plateaus (see left side of Fig. 6a). However, as the ridge spacing decreases to approximately 10 µm (line index 30), the honeycomb pattern is disrupted, and cells increasingly align along the ridges. At this scale, the structured surface prevents the formation of honeycomb structures, suggesting a physical limitation on cell–cell interactions due to spatial constraints. This can be seen in greater detail in the insets of Fig. 6b–d, showing how wide ridges display a honeycomb-shaped gap, while narrow ridges result in small groupings of cells. Furthermore, in the insets of Fig. 6b–d, the high resolution of the image is evident, clearly distinguishing the cells on both the ridges and the valleys.

In Fig. 6e, the orientation map shows a predominance of cyan, blue, and green colors, indicating an orientation trend around 0°. Figure 6f displays the orientation distribution for both populations, where the plateau population shows a preferred orientation of ±45°, with a maximum at 0°. In contrast, the valley population exhibits a more uniform orientation distribution, with a sharp peak centered at 0°, more sharp than in the plateau population. This result suggests that due to the pattern orientation, bacteria tend to have a preferred alignment parallel to the plateau lines. As shown in Fig. 6g, there is a height difference of 1.3 µm between the valley and plateau regions, yet the model still detects both bacterial populations with reasonable precision.

Figure 6h shows the bacterial density of both populations as a function of the line index. Bacterial density remains constant up to approximately line 60, corresponding to a plateau width of 5 µm; beyond this point, there is a sharp decline in bacterial density on the plateau. This finding provides valuable information for optimizing surfaces to prevent bacterial attachment and growth. To rule out drying or evaporation artifacts as the cause of this trend, we conducted a control experiment using gold nanoparticles deposited on the same patterned silicon oxide substrate. As shown in Supplementary Fig. 9, nanoparticle distribution remained homogeneous across different plateau widths, confirming that the observed bacterial density variations are not due to sample preparation artifacts. Lastly, it should be noted that no dependence on pattern geometry was observed in the other feature maps (see Supplementary Fig. 10).

As demonstrated here, this automated large-area AFM approach allows for high-throughput exploration of combinatorial surface libraries, where large areas can be scanned to assess how specific surface features, with varied spacing, height, shape or chemical modification, affect biofilm formation. By automating imaging and analysis across these structured gradients, we can efficiently identify configurations that reduce microbial adhesion or alter biofilm architecture, offering valuable insights for antifouling strategies. Additionally, this method can be extended to test other structural designs and materials, providing a robust platform for optimizing surface modifications in antifouling applications across diverse fields. Although this study primarily focuses on single-layer biofilm imaging, our approach can be readily extended to investigate three-dimensional biofilm structures with complex microarchitectures. Moreover, by adapting our methodology to cross-sectional studies, this technique could provide valuable insights into cellular arrangement and spatial organization within mature biofilms. In our observations, we detected the presence of cells on top of the first layer at longer time points, though only minimally, as shown in Supplementary Fig. 1.2. Recent work by Dayton et al.^72^ has demonstrated how cellular architecture within biofilms influences metabolic activity and antibiotic tolerance, further highlighting the importance of developing tools that enable detailed structural analysis across biofilm depth.

## Discussion

This work presents an automated platform for large-area AFM imaging, specifically designed to study biofilm growth and structure. By integrating high-resolution AFM with advanced image stitching algorithms, we mapped extensive regions of surface-attached *Pantoea* sp. YR343 cells, revealing detailed cellular and subcellular features as well as the overall community architecture. The surface-attached bacterial communities exhibit a characteristic honeycomb morphology on hydrophobic surfaces, with flagella bridging gaps between cell clusters, likely enhancing stability and propagation. High-resolution imaging provided insights into fine structures like flagella and pili, which are essential for understanding biofilm formation mechanisms. Additionally, we designed a patterned surface to examine how surface morphology influences bacterial attachment, on structured interfaces uncovering that bacterial density remains stable up to a plateau width of 5 µm, beyond which there is a sharp drop in the number of attached cells and a “break” in bacterial clusters. This observation provides valuable insights for designing antibacterial surfaces by controlling specific feature dimensions to inhibit cell attachment and biofilm formation. Accurate image stitching minimized distortions, preserving spatial relationships and enabling reliable extraction of quantitative descriptors such as cell count, confluency, and the creation of descriptor maps.

In the future, we plan to expand this platform to study living cells in liquid environments, capturing functional information about the nanomechanical properties such as elasticity and adhesion forces. To further accelerate data acquisition and reduce costs, we plan to implement a combination of sparse sampling and adaptive imaging paths over large areas. This approach will integrate low-resolution optical imaging with strategically selected, high-resolution AFM imaging, guided by a Bayesian framework. By focusing on high-resolution imaging only where needed, we aim to maximize efficiency and minimize resource expenditure. These advancements are expected to enhance our understanding of biofilm assembly dynamics and support more effective biofilm management strategies, particularly in clinical and industrial contexts. Continued integration of these techniques with machine learning and computational tools will further improve the efficiency and accuracy of biofilm research, driving significant progress in the field.

## Methods

### Cell culture

*Pantoea* sp. YR343 and ΔfliR mutant were grown in MOPS minimal media^73^ supplemented with 0.4% glucose overnight with shaking at 30 °C. Overnight cultures were re-inoculated and grown to an early exponential phase and adjusted to an optical density (OD, 600 nm) of 0.1 by diluting in MOPS media with 0.4% glucose. 8 mL of bacterial culture was added to a petri dish containing a silanized glass slide and incubated at 30 °C for a time the specified times prior to collection for imaging. At collection, the slide was carefully rinsed in three aliquots of Nanopure water, being dipped ten times in each aliquot, without being dried at any point. The slides were then fixed in 4% paraformaldehyde for 15 min and washed once more. Finally, the slides were dried with pressurized nitrogen.

The ΔfliR mutant of *Pantoea* sp. YR343 was generated following previously established protocols^56^. To construct the mutant, a 1000 bp flanking region upstream and downstream of the fliR gene was amplified and cloned into the pK18mobsacB plasmid. This construct was introduced into *Pantoea* sp. YR343 via electroporation and colonies were selected on kanamycin (50 μg/mL) to identify integrants. To promote homologous recombination and plasmid loss, selected colonies were passaged in rich media without antibiotic selection for three generations. Mutant candidates were then screened for motility loss, a hallmark of flagella-deficient strains, and their genotypes were confirmed using PCR analysis.

### Sample preparation and surface treatment

Avantor 25 mm × 25 mm Borosilicate glass coverslips were oxidative plasma cleaned in a vacuum chamber for 5 min, then transferred to a glass dish holding a container of 20 μL liquid trichloro(1H,1H,2H,2H-perfluorooctyl) silane (PFOTS). The coverslips were functionalized with PFOTS by vapor deposition at 80 °C for 60 min. Silanized glass coverslips were stored at room temperature until incubation with cell cultures.

### AFM measurements

The AFM images were acquired using the DriveAFM system from Nanosurf, a device equipped with a motorized stage that enables the scanning of large areas (>10 mm²), ideal for detailed studies of extensive bacterial biofilms. The microscope was controlled via a custom Python-based graphical interface that interacts directly with the microscope’s software, allowing users to define and automate grid scans with overlapping frames to capture large biofilm regions seamlessly.

For imaging, Multi75-G tips (Budget Sensors) were used for standard scans, while diamond tips AD-2.8-AS (Adama) were employed for high-resolution imaging, as they provide superior image quality and increased durability. All scans were performed in tapping mode with amplitude modulation, maintaining a setpoint of 60%. The grid scans were set with a 10% overlap between adjacent frames with a lateral size between 70 and 90 µm, a configurable parameter in the interface that facilitated large-area imaging and stitching process.

## Supplementary information

Supplementary information

## Data Availability

Data sets generated during the current study are available from the corresponding author on reasonable request.

## Code availability

The custom Large-Area-AFM Python package, developed for automated data acquisition and subsequent analysis, is publicly available on GitHub at https://github.com/Rmillansol/Large-Area-AFM-Analysis-and-Control.

## References

[1] López, D., Vlamakis, H. & Kolter, R. Biofilms. *Cold Spring Harb. Perspect. Biol.* **2**, a000398 (2010). 20519345 10.1101/cshperspect.a000398 PMC2890205

[2] Donlan, R. M. Biofilms: microbial life on surfaces. *Emerg. Infect. Dis.* **8**, 881 (2002). 12194761 10.3201/eid0809.020063 PMC2732559

[3] Abebe, G. M. The role of bacterial biofilm in antibiotic resistance and food contamination. *Int. J. Microbiol.* **2020**, 1705814 (2020). 32908520 10.1155/2020/1705814 PMC7468660

[4] Zhang, Y., Cai, Y., Zhang, B. & Zhang, Y.-H. P. J. Spatially structured exchange of metabolites enhances bacterial survival and resilience in biofilms. *Nat. Commun.* **15**, 7575 (2024). 39217184 10.1038/s41467-024-51940-3 PMC11366000

[5] Wimpenny, J., Manz, W. & Szewzyk, U. Heterogeneity in biofilms. *FEMS Microbiol. Rev.* **24**, 661–671 (2000). 11077157 10.1111/j.1574-6976.2000.tb00565.x

[6] Zhao, A., Sun, J. & Liu, Y. Understanding bacterial biofilms: from definition to treatment strategies. *Front. Cell. Infect. Microbiol.* **13**, 1137947 (2023). 37091673 10.3389/fcimb.2023.1137947 PMC10117668

[7] Dang, H. & Lovell, C. R. Microbial surface colonization and biofilm development in marine environments. *Microbiol. Mol. Biol. Rev.* **80**, 91–138 (2016). 26700108 10.1128/MMBR.00037-15 PMC4711185

[8] Azeredo, J. et al. Critical review on biofilm methods. *Crit. Rev. Microbiol.* **43**, 313–351 (2017). 27868469 10.1080/1040841X.2016.1208146

[9] Cleaver, L. & Garnett, J. A. How to study biofilms: technological advancements in clinical biofilm research. *Front. Cell. Infect. Microbiol.* **13**, 1335389 (2023). 38156318 10.3389/fcimb.2023.1335389 PMC10753778

[10] Wilson, C. et al. Quantitative and qualitative assessment methods for biofilm growth: a mini-review. *Res. Rev. J. Eng. Technol*. **6** (2017). PMC6133255 30214915

[11] Relucenti, M. et al. Microscopy methods for biofilm imaging: focus on SEM and VP-SEM pros and cons. *Biology* **10**, 51 (2021). 33445707 10.3390/biology10010051 PMC7828176

[12] Lozano, H. et al. Electrical properties of outer membrane extensions from *Shewanella oneidensis* MR-1. *Nanoscale* **13**, 18754–18762 (2021). 34747424 10.1039/d1nr04689f

[13] Lidke, D. S. & Lidke, K. A. Advances in high-resolution imaging–techniques for three-dimensional imaging of cellular structures. *J. Cell Sci.* **125**, 2571–2580 (2012). 22685332 10.1242/jcs.090027 PMC3706075

[14] Alldritt, B. et al. Automated structure discovery in atomic force microscopy. *Sci. Adv.* **6**, eaay6913 (2020). 32133405 10.1126/sciadv.aay6913 PMC7043916

[15] Binnig, G., Quate, C. F. & Gerber, C. Atomic force microscope. *Phys. Rev. Lett.* **56**, 930 (1986). 10033323 10.1103/PhysRevLett.56.930

[16] Alonso, J. L. & Goldmann, W. H. Feeling the forces: atomic force microscopy in cell biology. *Life Sci.* **72**, 2553–2560 (2003). 12672501 10.1016/s0024-3205(03)00165-6

[17] Botet-Carreras, A. et al. On the uptake of cationic liposomes by cells: from changes in elasticity to internalization. *Colloids Surf. B: Biointerfaces* **221**, 112968 (2023). 36335823 10.1016/j.colsurfb.2022.112968

[18] Millan-Solsona, R., Checa, M., Fumagalli, L. & Gomila, G. Mapping the capacitance of self-assembled monolayers at metal/electrolyte interfaces at the nanoscale by in-liquid scanning dielectric microscopy. *Nanoscale* **12**, 20658–20668 (2020). 33043923 10.1039/d0nr05723a

[19] Ahimou, F., Semmens, M. J., Novak, P. J. & Haugstad, G. Biofilm cohesiveness measurement using a novel atomic force microscopy methodology. *Appl. Environ. Microbiol.* **73**, 2897–2904 (2007). 17337563 10.1128/AEM.02388-06 PMC1892862

[20] Bremer, P. J., Geese, G. G. & Drake, B. Atomic force microscopy examination of the topography of a hydrated bacterial biofilm on a copper surface. *Curr. Microbiol.* **24**, 223–230 (1992).

[21] Chatterjee, S., Biswas, N., Datta, A., Dey, R. & Maiti, P. Atomic force microscopy in biofilm study. *Microscopy* **63**, 269–278 (2014). 24793174 10.1093/jmicro/dfu013

[22] Bolshakova, A. V., Kiselyova, O. I. & Yaminsky, I. V. Microbial surfaces investigated using atomic force microscopy. *Biotechnol. Prog.* **20**, 1615–1622 (2004). 15575691 10.1021/bp049742c

[23] Limoli, D. H., Jones, C. J. & Wozniak, D. J. Bacterial extracellular polysaccharides in biofilm formation and function. *Microb. Biofilms* 223–247 (2015). 10.1128/microbiolspec.MB-0011-2014 PMC4657554 26185074

[24] Krieg, M. et al. Atomic force microscopy-based mechanobiology. *Nat. Rev. Phys.* **1**, 41–57 (2019).

[25] Checa, M., Millan-Solsona, R., Glinkowska Mares, A., Pujals, S. & Gomila, G. Dielectric imaging of fixed HeLa cells by in-liquid scanning dielectric force volume microscopy. *Nanomaterials* **11**, 1402 (2021). 34070690 10.3390/nano11061402 PMC8226567

[26] Di Muzio, M. et al. Dielectric properties and lamellarity of single liposomes measured by in-liquid scanning dielectric microscopy. *J. Nanobiotechnol.* **19**, 167 (2021). 10.1186/s12951-021-00912-6 PMC8176598 34082783

[27] Balakrishnan, H. et al. Depth mapping of metallic nanowire polymer nanocomposites by scanning dielectric microscopy. *Nanoscale* **13**, 10116–10126 (2021). 34060583 10.1039/d1nr01058a

[28] Muzio, M. D., Millan-Solsona, R., Borrell, J. H., Fumagalli, L. & Gomila, G. Cholesterol effect on the specific capacitance of submicrometric DOPC bilayer patches measured by in-liquid scanning dielectric microscopy. *Langmuir* **36**, 12963–12972 (2020). 33084346 10.1021/acs.langmuir.0c02251

[29] Boschker, H. T. et al. Efficient long-range conduction in cable bacteria through nickel protein wires. *Nat. Commun.* **12**, 3996 (2021). 34183682 10.1038/s41467-021-24312-4 PMC8238962

[30] Müller, D. J. et al. Atomic force microscopy-based force spectroscopy and multiparametric imaging of biomolecular and cellular systems. *Chem. Rev.* **121**, 11701–11725 (2020). 33166471 10.1021/acs.chemrev.0c00617

[31] Kochan, K. et al. In vivo atomic force microscopy–infrared spectroscopy of bacteria. *J. R. Soc. Interface* **15**, 20180115 (2018). 29593091 10.1098/rsif.2018.0115 PMC5908543

[32] Checa, M. et al. Mapping the dielectric constant of a single bacterial cell at the nanoscale with scanning dielectric force volume microscopy. *Nanoscale* **11**, 20809–20819 (2019). 31657419 10.1039/c9nr07659j

[33] Van Der Hofstadt, M. et al. Internal hydration properties of single bacterial endospores probed by electrostatic force microscopy. *ACS Nano* **10**, 11327–11336 (2016). 28024372 10.1021/acsnano.6b06578

[34] Beech, I. B., Smith, J. R., Steele, A. A., Penegar, I. & Campbell, S. A. The use of atomic force microscopy for studying interactions of bacterial biofilms with surfaces. *Colloids Surf. B: Biointerfaces* **23**, 231–247 (2002).

[35] Gingichashvili, S., Feuerstein, O. & Steinberg, D. Topography and expansion patterns at the biofilm–agar interface in *Bacillus subtilis* biofilms. *Microorganisms* **9**, 84 (2020). 33396528 10.3390/microorganisms9010084 PMC7823598

[36] Masud, N., Rade, J., Hasib, M. H. H., Krishnamurthy, A. & Sarkar, A. Machine learning approaches for improving atomic force microscopy instrumentation and data analytics. *Front. Phys.* **12**, 1347648 (2024).

[37] Kalinin, S. V. et al. Automated and autonomous experiments in electron and scanning probe microscopy. *ACS Nano* **15**, 12604–12627 (2021). 34269558 10.1021/acsnano.1c02104

[38] Vasudevan, R. K. et al. Autonomous experiments in scanning probe microscopy and spectroscopy: choosing where to explore polarization dynamics in ferroelectrics. *ACS Nano* **15**, 11253–11262 (2021). 34228427 10.1021/acsnano.0c10239

[39] Liu, Y. et al. Experimental discovery of structure–property relationships in ferroelectric materials via active learning. *Nat. Mach. Intell.* **4**, 341–350 (2022).

[40] Chandrashekar, A., Belardinelli, P., Bessa, M. A., Staufer, U. & Alijani, F. Quantifying nanoscale forces using machine learning in dynamic atomic force microscopy. *Nanoscale Adv.* **4**, 2134–2143 (2022). 35601812 10.1039/d2na00011c PMC9063738

[41] Kocur, V., Hegrová, V., Patočka, M., Neuman, J. & Herout, A. Correction of AFM data artifacts using a convolutional neural network trained with synthetically generated data. *Ultramicroscopy* **246**, 113666 (2023). 36599269 10.1016/j.ultramic.2022.113666

[42] Kelley, K. P. et al. Fast scanning probe microscopy via machine learning: non-rectangular scans with compressed sensing and gaussian process optimization. *Small* **16**, 2002878 (2020). 10.1002/smll.202002878 32780947

[43] Checa, M. et al. High-speed mapping of surface charge dynamics using sparse scanning Kelvin probe force microscopy. *Nat. Commun.* **14**, 7196 (2023). 37938577 10.1038/s41467-023-42583-x PMC10632481

[44] Alldritt, B. et al. Automated tip functionalization via machine learning in scanning probe microscopy. *Comput. Phys. Commun.* **273**, 108258 (2022).

[45] Liu, Y. et al. AEcroscopy: a software–hardware framework empowering microscopy toward automated and autonomous experimentation. *Small Methods* **8**, 2301740 (2024). 10.1002/smtd.202301740 38639016

[46] Liu, Y., Checa, M. & Vasudevan, R. K. Synergizing human expertise and AI efficiency with language model for microscopy operation and automated experiment design*. *Mach. Learn.: Sci. Technol.* **5**, 02LT01 (2024).

[47] Krull, A., Hirsch, P., Rother, C., Schiffrin, A. & Krull, C. Artificial-intelligence-driven scanning probe microscopy. *Commun. Phys.* **3**, 54 (2020).

[48] Sokolov, I. On machine learning analysis of atomic force microscopy images for image classification, sample surface recognition. *Phys. Chem. Chem. Phys.* **26**, 11263–11270 (2024). 38477533 10.1039/d3cp05673b PMC11182436

[49] O'Dowling, A. T., Rodriguez, B. J., Gallagher, T. K. & Thorpe, S. D. Machine learning and artificial intelligence: enabling the clinical translation of atomic force microscopy-based biomarkers for cancer diagnosis. *Comput. Struct. Biotechnol. J.* **24**, 661–671 (2024). 39525667 10.1016/j.csbj.2024.10.006 PMC11543504

[50] Rade, J. et al. Deep learning for live cell shape detection and automated afm navigation. *Bioengineering* **9**, 522 (2022). 36290490 10.3390/bioengineering9100522 PMC9598706

[51] Petrov, M. & Sokolov, I. Machine learning allows for distinguishing precancerous and cancerous human epithelial cervical cells using high-resolution AFM imaging of adhesion maps. *Cells* **12**, 2536 (2023). 37947614 10.3390/cells12212536 PMC10650179

[52] Tang, B. et al. Machine learning-aided atomic structure identification of interfacial ionic hydrates from AFM images. *Natl Sci. Rev.* **10**, nwac282 (2023). 37266561 10.1093/nsr/nwac282 PMC10232042

[53] Rahman Laskar, M. A. & Celano, U. Scanning probe microscopy in the age of machine learning. *APL Mach. Learn.* **1**, 041501 (2023).

[54] Bible, A. N. et al. A carotenoid-deficient mutant in *Pantoea* sp. YR343, a bacteria isolated from the rhizosphere of *Populus deltoides*, is defective in root colonization. *Front. Microbiol.* **7**, 491 (2016). 27148182 10.3389/fmicb.2016.00491 PMC4834302

[55] Bible, A. N., Chang, M. & Morrell-Falvey, J. L. Identification of a diguanylate cyclase expressed in the presence of plants and its application for discovering candidate gene products involved in plant colonization by Pantoea sp. YR343. *PLoS ONE* **16**, e0248607 (2021). 34288916 10.1371/journal.pone.0248607 PMC8294551

[56] Halsted, M. C., Bible, A. N., Morrell-Falvey, J. L. & Retterer, S. T. Quantifying biofilm propagation on chemically modified surfaces. *Biofilm* **4**, 100088 (2022). 36303845 10.1016/j.bioflm.2022.100088 PMC9594113

[57] Wang, C.-W., Ka, S.-M. & Chen, A. Robust image registration of biological microscopic images. *Sci. Rep.* **4**, 6050 (2014). 25116443 10.1038/srep06050 PMC4131219

[58] Pellikka, M. & Lahtinen, V. A robust method for image stitching. *Pattern Anal. Appl.* **24**, 1847–1858 (2021).

[59] Chalfoun, J. et al. MIST: accurate and scalable microscopy image stitching tool with stage modeling and error minimization. *Sci. Rep.* **7**, 4988 (2017). 28694478 10.1038/s41598-017-04567-y PMC5504007

[60] D. G. Lowe, Object recognition from local scale-invariant features, *Proceedings of the Seventh IEEE International Conference on Computer Vision*, Kerkyra, Greece, vol. 2, pp. 1150–1157 (1999).

[61] Karami, E., Prasad, S. & Shehata, M. Image matching using SIFT, SURF, BRIEF and ORB: performance comparison for distorted images. arXiv preprint arXiv:1710.02726 (2017).

[62] Mohammadi, F. S., Mohammadi, S. E., Mojarad Adi, P., Mirkarimi, S. M. A. & Shabani, H. A comparative analysis of pairwise image stitching techniques for microscopy images. *Sci. Rep.* **14**, 9215 (2024). 38649426 10.1038/s41598-024-59626-y PMC11035624

[63] Ricci, D. & Braga, P. C. Recognizing and avoiding artifacts in AFM imaging. In *Atomic Force Microscopy: Biomedical Methods and Applications* vol 242, 25–37 (Humana Press, 2004). 10.1385/1-59259-647-9:25 14578511

[64] Minaee, S. et al. Image segmentation using deep learning: a survey. *IEEE Trans. Pattern Anal. Mach. Intell.* **44**, 3523–3542 (2021). 10.1109/TPAMI.2021.3059968 33596172

[65] Ragab, M. G. et al. A comprehensive systematic review of YOLO for medical object detection (2018 to 2023). *IEEE Access, vol. 12, pp. 57815-57836*10.1109/ACCESS.2024.3386826 (2024).

[66] Sohan, M., Sai Ram, T., Reddy, R. & Venkata, C. In *International Conference on Data Intelligence and Cognitive Informatics* 529–545. Algorithms for Intelligent Systems. Springer, Singapore. 10.1007/978-981-99-7962-2_39.

[67] Wahid, F., Ma, Y., Khan, D., Aamir, M. & Bukhari, S. U. Biomedical image segmentation: a systematic literature review of deep learning based object detection methods. arXiv preprint arXiv:2408.03393 (2024).

[68] Rayed, M. E. et al. Deep learning for medical image segmentation: State-of-the-art advancements and challenges. *Inform. Med. Unlocked.* **47**, 101504 (2024).

[69] Roboflow Roboflow: Annotate, train, and deploy computer vision models (Version 1.0) https://roboflow.com (2024).

[70] Ahmad Khalili, A. & Ahmad, M. R. A review of cell adhesion studies for biomedical and biological applications. *Int. J. Mol. Sci.* **16**, 18149–18184 (2015). 26251901 10.3390/ijms160818149 PMC4581240

[71] Van Gerven, N., Waksman, G. & Remaut, H. Pili and flagella: biology, structure, and biotechnological applications. *Prog. Mol. Biol. Transl. Sci.* **103**, 21–72 (2011). 21999994 10.1016/B978-0-12-415906-8.00005-4

[72] Dayton, H. et al. Cellular arrangement impacts metabolic activity and antibiotic tolerance in *Pseudomonas aeruginosa* biofilms. *PLoS Biol.* **22**, e3002205 (2024). 38300958 10.1371/journal.pbio.3002205 PMC10833521

[73] Neidhardt, F. C., Bloch, P. L. & Smith, D. F. Culture medium for enterobacteria. *J. Bacteriol.* **119**, 736–747 (1974). 4604283 10.1128/jb.119.3.736-747.1974 PMC245675

