# Supplementary material for: Analysis of biofilm assembly by large area automated AFM
Source: NPJ Biofilms Microbiomes. 2025 May 8;11:75. doi: 10.1038/s41522-025-00704-y (PMC12062311; doi:10.1038/s41522-025-00704-y)
Supplement: Supplementary file 1 — Supplementary information [file 41522_2025_704_MOESM1_ESM.pdf]

The United States Government retains and the publisher, by accepting the article for publication, acknowledges that the United States Government retains a nonexclusive, paid-up, irrevocable, worldwide license to publish or reproduce the published form of this manuscript, or allow others to do so, for the United States Government purposes. The Department of Energy will provide public access to these results of federally sponsored research in accordance with the DOE Public Access Plan (*http://energy.gov/downloads/doe-public-access-plan*).

Supplementary information

Ruben Millan-Solsona,[1*] Spenser R. Brown,[2] Lance Zhang,[2] Sita Sirisha Madugula,[1] HuanHuan Zhao,[3] Blythe Dumerer, [1] Amber N. Bible,[2] Nickolay V. Lavrik,[1] Rama K. Vasudevan,[1] Arpan Biswas,[4] Jennifer L. Morrell-Falvey,[2] Scott Retterer, [1,2] Martí Checa,[1] Liam Collins.[1*]

[1]Center for Nanophase Materials Sciences, Oak Ridge National Laboratory, Oak Ridge, Tennessee 37831, USA.
[2]Biosciences Division, Oak Ridge National Laboratory, Oak Ridge, Tennessee 37831, USA.
[3]Bredesen Center for Interdisciplinary Research, University of Tennessee, Knoxville, USA
[4]University of Tennessee-Oak Ridge Innovation Institute, Knoxville, TN 37996, USA.

Email: solsonarm@ornl.gov & collinslf@ornl.gov

Supplementary section 1: AFM Validation of Flagellar Structures and Imaging in Liquid Using a Control Strain

To distinguish whether the dendritic structures observed in the wild-type strain were flagella or another appendage, such as fimbriae, we conducted experiments using a ΔfliR mutant of *Pantoea* sp. YR343. Since the *fliR* gene is essential for flagellar assembly, we hypothesized that if these structures were indeed flagella, they would be absent in the mutant due to impaired motility and flagellar biosynthesis. Our results confirmed this hypothesis—the dendritic structures observed in wild-type cells were absent in the ΔfliR mutant, supporting their identification as flagella. This finding is consistent with *Pantoea*'s peritrichous flagella arrangement, where

multiple flagella extend outward from the bacterial surface. Additionally, to ensure that our methodology accurately captures bacterial surface features under physiological conditions, we conducted AFM imaging in liquid. Together, these validations reinforce the robustness of our imaging approach and confirm the identity of the observed structures as flagella.

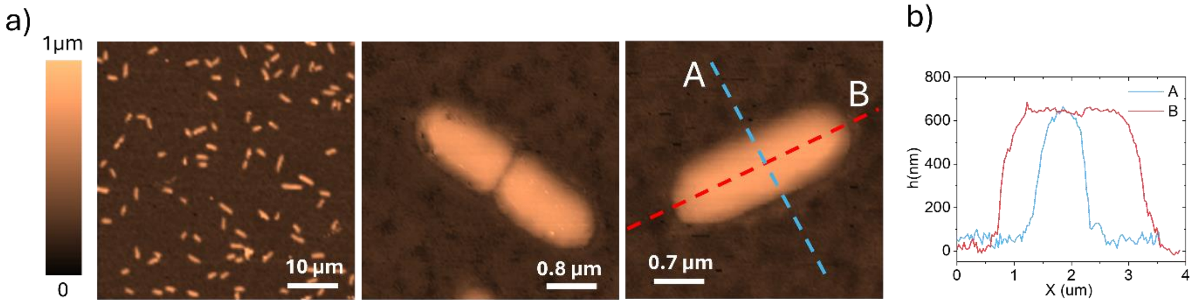

Supplementary Figure 1.1. *AFM analysis of the ΔfliR mutant of Pantoea sp. YR343. (a) AFM images of the ΔfliR mutant in physiological medium, showing the absence of dendritic structures observed in the wild-type strain. b) Corresponding topographic profiles taken along the marked regions in (a), confirming the lack of flagellar appendages.*

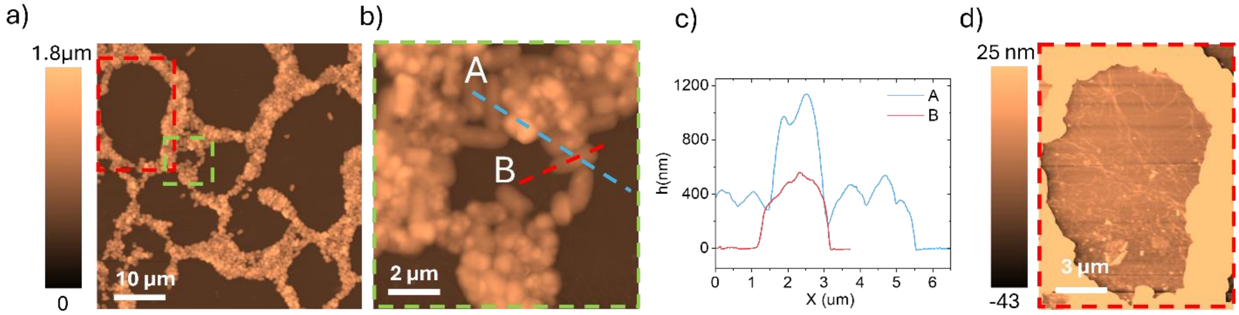

Supplementary Figure 1.2. *In (a, b, d), an image of Pantoea sp. YR343 rehydrated on a PFOTS-coated silicon substrate is shown. The image was acquired in a physiological medium (PBS 1x) using the DriveAFM. In (b), a zoomed-in view of the green-outlined region in (a) reveals*

two distinct layers of bacterial cells. In (c), two topographic profiles corresponding to the dashed lines A and B in (b) clearly show bacteria positioned above the first bacterial layer. Finally, in (d), the substrate corresponding to the red-outlined region in (a) is highlighted, where bacterial flagella can be observed on the surface.

Supplementary section 2: Model Training and Data Augmentation

To enhance the accuracy and robustness of bacterial detection in AFM images, we trained a YOLOv8 model using experimental images with a size of $15 \times 15\ \mu m^2$. The training process was performed on the Roboflow platform, where a curated dataset of experimentally acquired images was used. To expand this dataset, we employed data augmentation techniques, essential for improving the model's ability to generalize across various bacterial orientations and positions. In Supplementary Figure 2, an example of the data augmentation process is presented, specifically showcasing image rotation. By rotating the original images, we created variations that simulate different bacterial orientations, thus helping the model recognize bacteria more reliably in diverse scenarios. This augmentation is a key step in maximizing the training data without needing additional experimental images, enhancing the model's detection performance.

We would like to emphasize that the training dataset consisted of 80 manually labeled images of small areas, which were then augmented to a total of 352 images. These images do not overlap or correspond to any of the large-area images analyzed subsequently. The training images form a distinct dataset, independent of the dataset used to obtain the results presented.

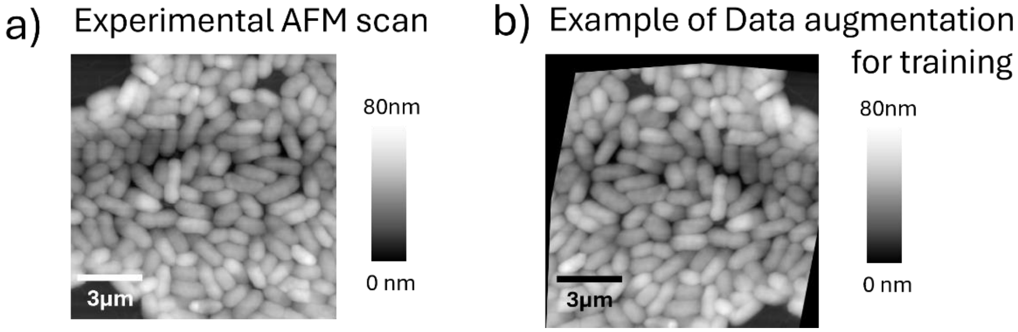

Supplementary Figure 2. *Data augmentation steps are ±90° rotations, ±10° shear (vertical and/or horizontal) and ±25% saturation. In (a) an original training image is shown and in (b) a rotated image for data augmentation.*

Supplementary section *3*: Detection Accuracy and Confidence Parameter

The detection precision of bacteria using YOLOv8 is highly influenced by the confidence threshold, a hyperparameter that determines the model's certainty in identifying an object as a bacterium. Adjusting this threshold allows us to balance between high precision and the risk of false positives (detecting non-bacterial structures as bacteria). Supplementary Figure 3 illustrates the relationship between the confidence threshold and detection accuracy. It highlights that while a lower confidence threshold may capture more bacteria, it can also increase the likelihood of erroneous detections. Conversely, a higher confidence threshold can yield higher precision but may miss some bacteria. Therefore, optimizing the confidence parameter is crucial to ensure adequate precision without compromising specificity.

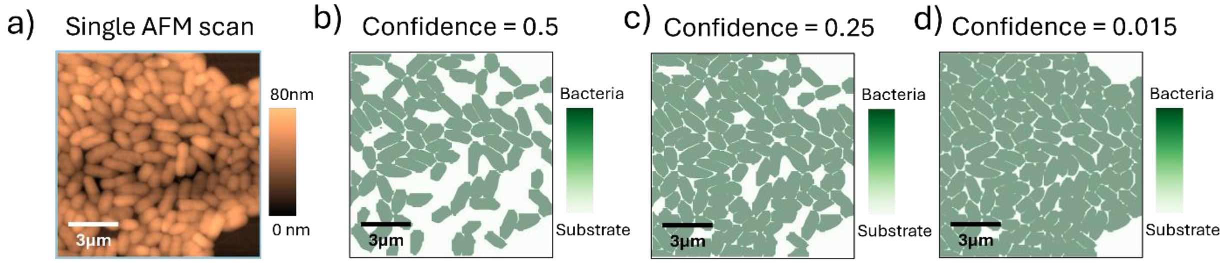

Supplementary Figure 3. *(a) Single AFM scan and precision of bacteria detection with the confidence hyperparameter, for three different values (b) 0.5, (c) 0.25 and (d) 0.015.*

Supplementary section 4: Quantitative segmentation

As an example data set, in Supplementary Figure 4, the larger stiched AFM image is shown as well as zoomed-in region reveals more clearly the individual bacterial cells targeted for segmentation and analysis. We find that using YOLOv8, the segmentation process accurately identifies the boundaries of each bacterium, as seen in the green-colored segmented image (Supplementary Figure 4.b), where the bacterial surface coverage is measured at 85.23%. The corresponding data table (Supplementary Figure 4.c) presents quantitative metrics for each bacterium, including key features such as area, perimeter, orientation, centroid coordinates, eccentricity, and cell count. Overall, this workflow enables automated and comprehensive analysis of community structure, providing high-resolution metrics at the cellular level and facilitating a deeper understanding of biofilm morphology and growth patterns. [4]

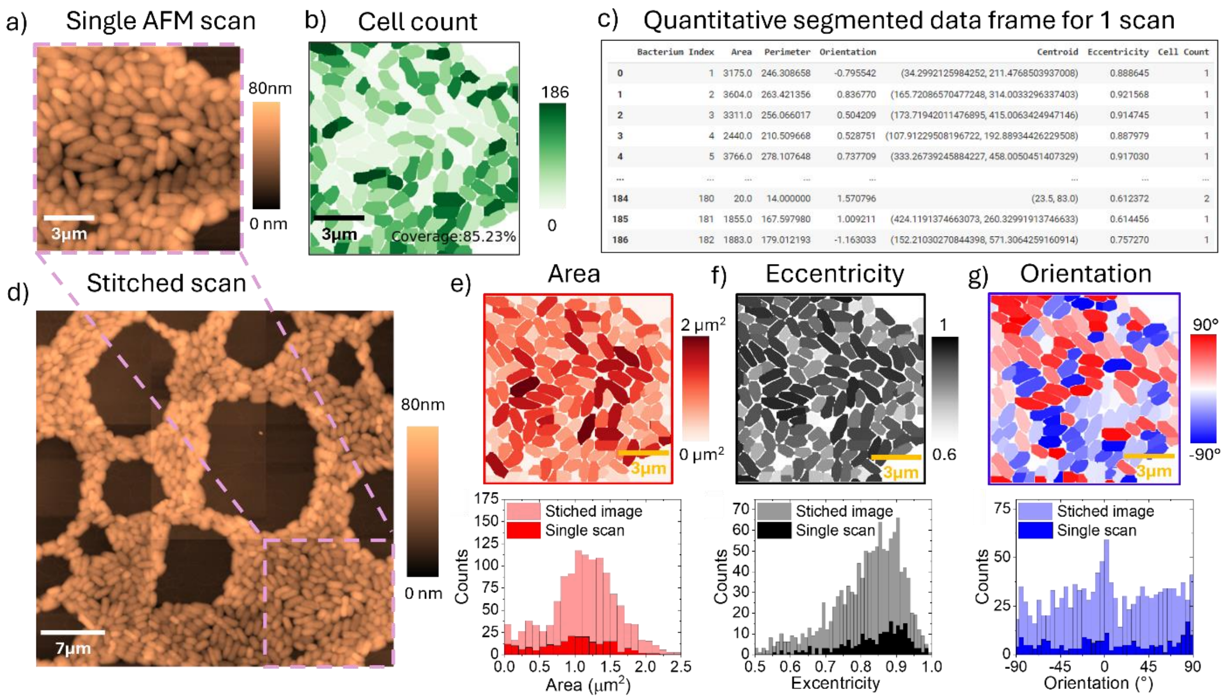

Supplementary Figure 4. *An example dataset is shown. (a) Original AFM image. (b) Detection and counting of bacteria. (c) table obtained with the quantification of various characteristics extracted from the segmentation, such as area, eccentricity, major axis, minor axis, etc. In (d) a large stitched area is shown. From (e-g) are the area, eccentricity and orientation maps for the region shown in (a). Below in (e-g) the distribution is shown for the individual maps and for the entire stitched area shown in (d).*

Supplementary section 5: Automated analysis of bacterial surface attachment using Large-Area AFM data

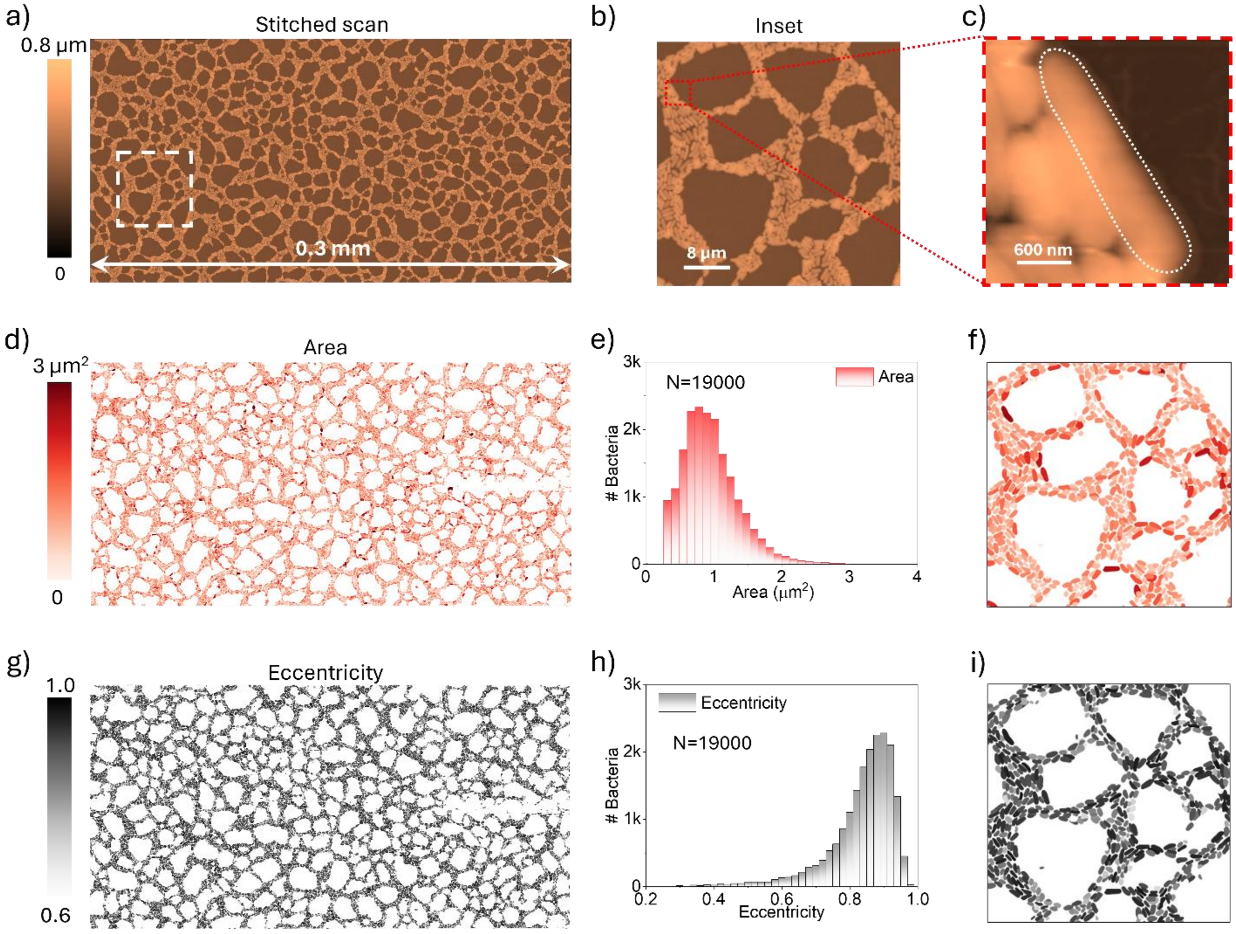

Supplementary Figure 5*. (a) Large area stitched topographic image of Pantoea sp. YR343 cells grown on PFOTS-treated glass surfaces for 60 minutes. (b) A zoom of the region marked in (a) and in (c) a zoom of the dotted red box marked in (b) showing a very long bacterial cells likely in the process of cell division. (d-f) The Area map, statistics and a zoom of the area map of the same region marked in (a). (g-i) The eccentricity map, the statistics and a zoom of the eccentricity map of the same region marked in (a).*

Supplementary section 6: Cluster Analysis of cell Area

The spatial analysis of cell clustering employed two key techniques namely, Moran's I and K-means clustering, along with calculating the radially averaged 2D autocorrelations. Moran's I is a statistical measure of spatial autocorrelation that quantifies whether similar values (e.g., cell area) cluster together in space. A positive Moran's I indicates clustering of similar values, while a negative value suggests dissimilarity (e.g., large cells near small cells). In this study, we observed a weak but statistically significant positive Moran's I (0.0714, p-value = 0.001), indicating slight clustering of similarly sized cells across the analyzed region. To investigate the possibility of local clustering, K-means clustering, a machine learning technique, was used to group cells based on their sizes into distinct clusters. The results suggested some grouping of cells by area, reinforcing the presence of localized clustering patterns. The radial autocorrelation function further showed that spatial dependency diminishes rapidly with distance, revealing that clustering is limited to small scales, while patterns become random at larger distances. The radially averaged 2D autocorrelation, which integrates directional correlations into a single radial metric, supports these findings by highlighting the rapid decay of spatial dependency beyond small scales.

Although the observed correlations were weak, this type of spatial analysis can be highly informative in other contexts, such as identifying microbial colony development patterns, analyzing how resource gradients affect biofilm structure, or studying the effects of surface engineering on bacterial attachment for the design of antibacterial materials.

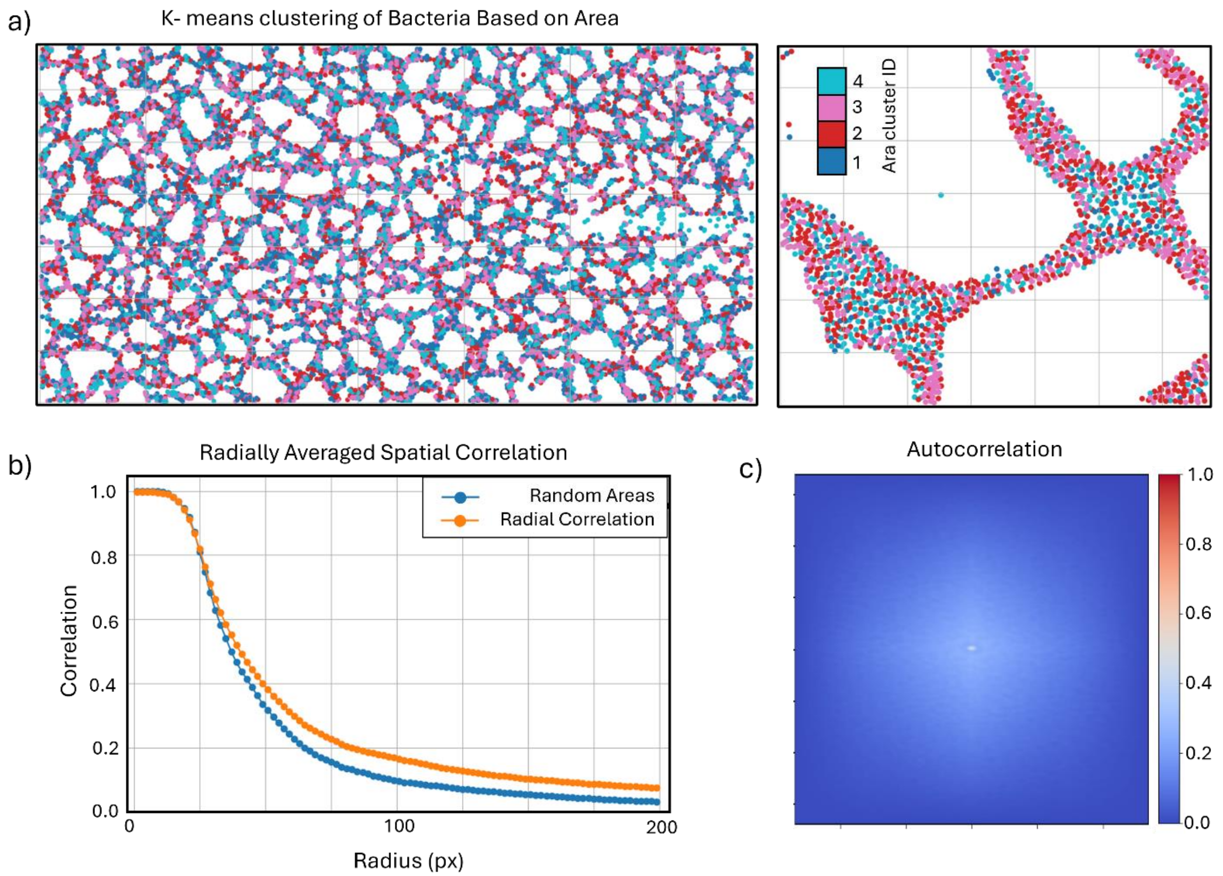

Supplementary Figure 6. *(a) Mapa de la distribucion de clusterbasados en el area mediante k-means para k=4, se observa claramente como la distribucion no es aleatoria. (b) Comparacion de autocorrelacion obserbada para la distribucion en (a) versus una distribucion random. (c) Se muestra la autocorrelacion de (a) debil pero estadisticamente significativa (p-value = 0.001).*

Supplementary section **7: Flagella analysis using Large-Area AFM data**

To create the stitched topographic image highlighting the flagella shown in Supplementary Figure 7.a, it was necessary to perform a specific flattening of each region of Figure 5.a. To do

this, a mask was made using a 150 nm threshold and the bacteria-free areas were selected. Then a segmentation by area has been carried out, for example in figure S6.b the segmentation of regions 1 to 19 is shown (each color represents a different region) corresponding to the dotted white box in figure S6.a. With the regions well delimited, we have proceeded to cut each of the regions and carry out a specific flattening for each region, greatly improving the flattening. For example, supplementary figure 7.c shows region 3 of supplementary figure 7.b (light pink) where we can see the original cropped region, the cropped mask showing the area of the gap, the flattened region, the mask to highlight the flagella and calculate the area covered by them and the area of the gap of each of the regions. Furthermore, as highlighted in the main text, it allows calculating the statistical dependence on height (Supplementary figure 7.e) and the relationship between the area covered by the flagella and the area of the gap.

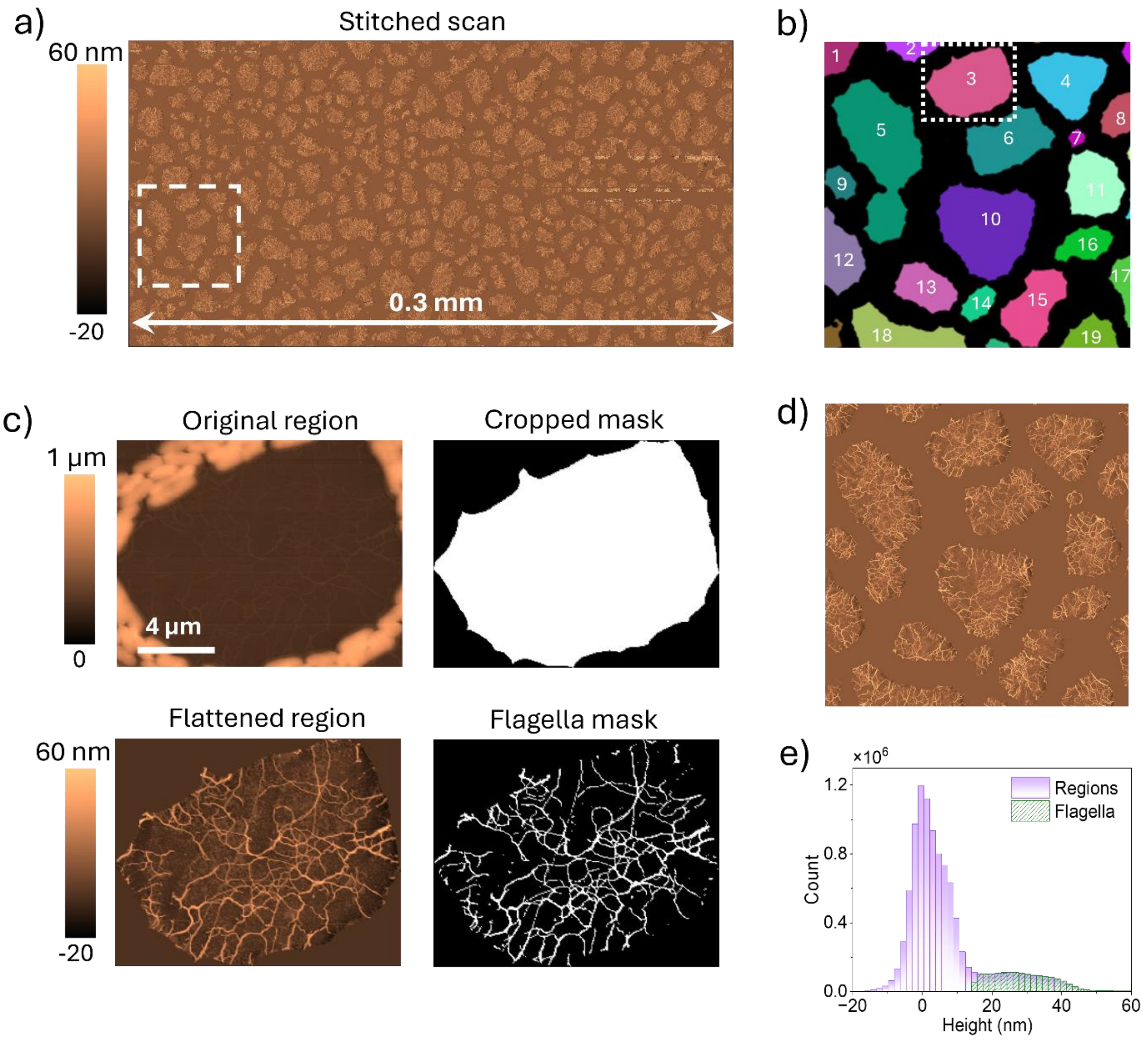

Supplementary Figure 7. *(a) Stitched topographic image highlighting the flagella. (b) Example of segmentation by regions corresponding to the dotted white box in (a). In (c) the cropped original region, the region mask area, the flattened region and the mask used to select the area covered by the flagella are shown. In (d) a zoom of the flattened regions corresponding to the dotted white box in (a) is shown and finally in (e) the height statistics corresponding to the entire image (a) (not counting the bacteria) and the flagella areas are shown.*

Supplementary section 8: Analytical model of flagella coverage

A simple model has been used to describe the relationship between the area enclosed by bacteria and the area covered by the flagella within that region. Suppose we have a circle of bacteria with radius $\boldsymbol{r}$, where each bacterium has an average length $\boldsymbol{l_b}$, enclosing the circle's area $\boldsymbol{A = \pi r^2}$. The number of bacteria on the perimeter $\boldsymbol{l = 2\pi r}$ is then given by:

$$\boldsymbol{n = \frac{l}{l_b}}$$

If we assume that each bacterium has $\boldsymbol{k}$ flagella and that each flagellum has a probability $\boldsymbol{p}$ of being inside the circle, the number of flagella inside the circle is:

$$\boldsymbol{n_f = nkp}$$

If we further assume that each flagellum covers an average area $\boldsymbol{a_f}$, the area covered by the flagella (A_f) inside the circle is given by:

$$\boldsymbol{A_f = n_f a_f}$$

Now, substituting $\boldsymbol{n_f}$ in terms of $\boldsymbol{n}$, and subsequently in terms of the circle's perimeter, we have:

$$\boldsymbol{A_f = nkpa_f = \frac{l}{l_b} kpa_f} \qquad \textbf{(1)}$$

The perimeter of the circle can also be expressed in terms of its area as follows:

$$\boldsymbol{l = 2\pi r = 2\pi\sqrt{\frac{A}{\pi}} = \frac{2}{\pi}\sqrt{A}}$$

Substituting the above expression for $\boldsymbol{l}$ into equation (1), we find that the area covered by the flagella is:

$$A_f = \frac{l}{l_b} kpa_f = \frac{2kpa_f}{\pi l_b}\sqrt{A}$$

Defining the constant term $\beta \equiv \frac{2kpa_f}{\pi l_b}$, the resulting relationship becomes:

$$A_f = \beta A^{1/2}$$

Thus, if only the flagella located on the perimeter contributed to the covered area inside the circle, we would expect a relationship where the covered area grows as the square root of the circle's area. However, in our case, we observe an exponent close to 0.9, indicating that not only the flagella at the perimeter contribute to the covered area, but also that more interior flagella are present within the studied areas.

Supplementary section 9: Automated analysis of community structure using Large-Area AFM data

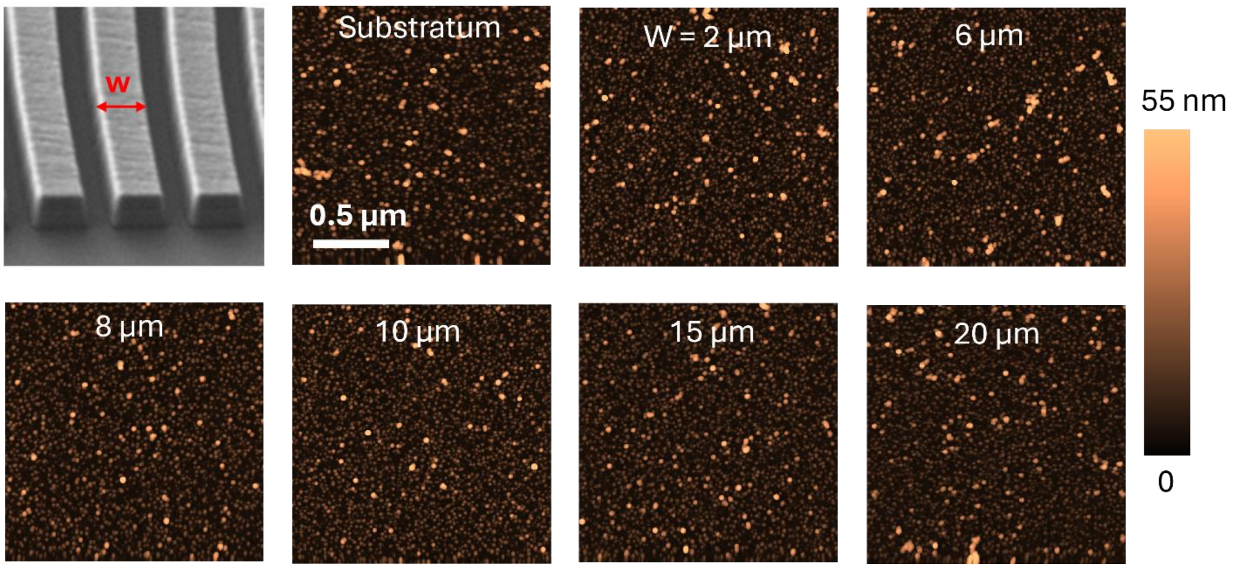

Supplementary Figure 9. *Control experiment to assess the impact of drying or evaporation artifacts. (Top left) Schematic representation of the patterned silicon oxide structure. (Right and*

bottom) AFM topographic images of gold nanoparticles deposited on the patterned surface, prepared using the same sample preparation protocol. The homogeneous distribution of nanoparticles across different line or mesa widths confirms that variations in bacterial attachment are not due to drying or evaporation effects.

Supplementary section 10: Feature maps

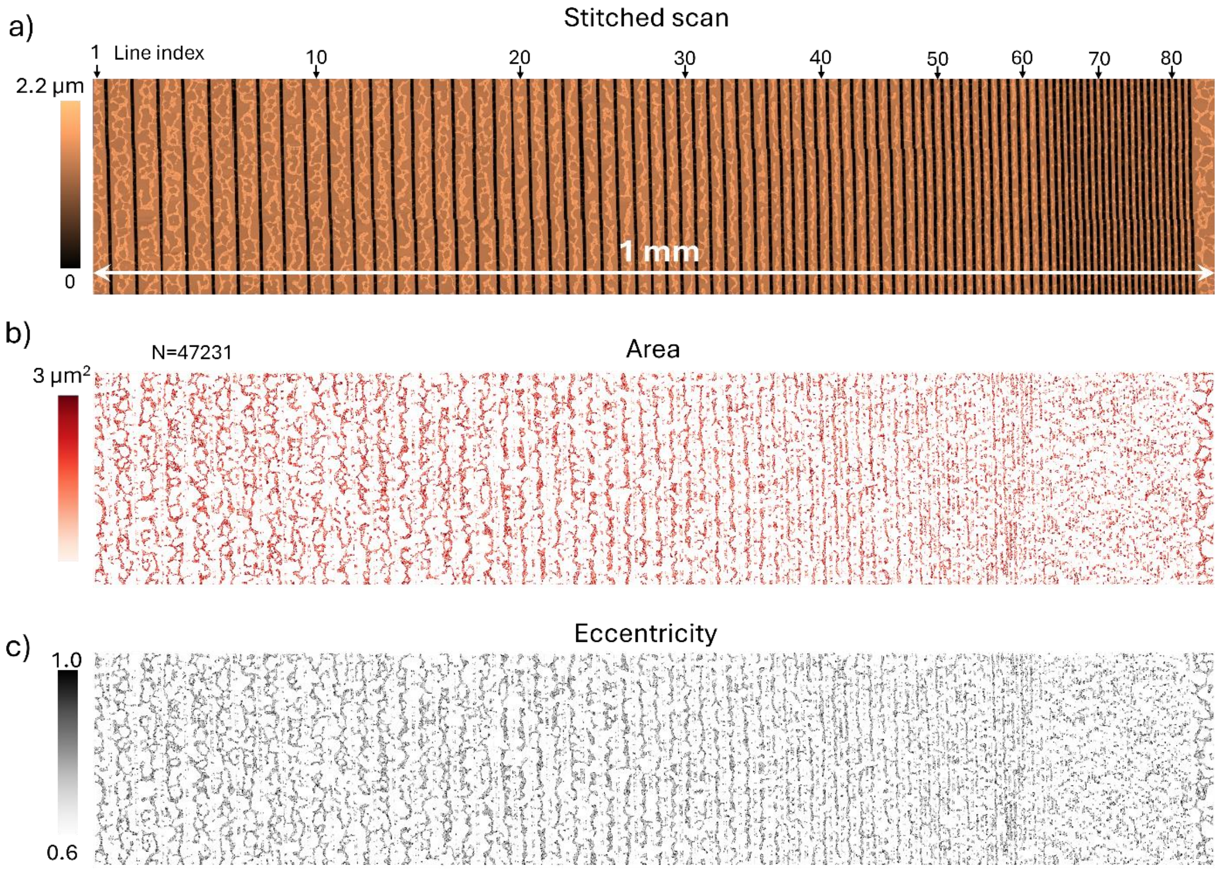

Supplementary Figure 10. *(a) Large area stitched image of bacterial cells grown on a patterned surface. (b) Area map and (c) eccentricity map are shown.*
